# Size dependent competitive adsorption of polystyrene micro and nanoplastics on a magnetic Fe_3_O_4_@MIL-100(Fe)/CNT hybrid: interfacial mechanisms and water treatment performance

**DOI:** 10.1039/d6ra07127a

**Published:** 2026-09-14

**Authors:** Meric Yilmaz Salman, Türker Salman, Mustafa Ersin Pekdemir

**Affiliations:** a Keban Vocational School, Keban Firat University Elazığ Türkiye meric.yilmaz@firat.edu.tr; b Department of Pharmacy Services, Kovancılar Vocational School, Firat University Elazığ Türkiye; c Polymer and Biomaterials Research Laboratory, Department of Chemistry, Faculty of Science, Firat University Elazığ Türkiye

## Abstract

The simultaneous capture of micro and nanoplastics is governed by particle size dependent transport and interfacial interactions that cannot be fully understood from single component experiments. Here, the competitive adsorption of 1 µm polystyrene microplastics (PS MPs) and 100 nm polystyrene nanoplastics (PS NPs) was investigated in single and binary component systems at the interface of a magnetically recoverable Fe_3_O_4_@MIL-100(Fe)/CNT hybrid (mFMC). In the single component system, PS MPs reached approximately 95% removal within 60 min, whereas PS NPs approached equilibrium more gradually. In the binary system, the presence of PS NPs markedly reduced the early stage adsorption rate of PS MPs, indicating size dependent competition at the heterogeneous mFMC-water interface. At pH 7, removal efficiencies reached 93.0% for PS MPs and 92.5% for PS NPs within 360 min. Zeta potential measurements and classical DLVO calculations provided a consistent framework for interpreting the pH dependent adsorption behavior. Interaction energy profiles under acidic and neutral conditions were consistent with high removal, whereas the energy barriers observed under alkaline conditions agreed with the decline in particle attachment. Raman spectral changes together with BET and SEM observations further supported surface coverage and possible contributions from hydrophobic and π–π interactions at CNT rich interfacial regions. The hybrid maintained removal efficiencies of at least 90% in tap water, textile wastewater and urban wastewater treatment plant effluent. After five adsorption–desorption cycles, removal remained at 90.2% for PS MPs and 88.0% for PS NPs. These findings establish a mechanistic link between particle size, competitive interfacial access and pH dependent interaction energies, providing a basis for the simultaneous removal of mixed size plastic contaminants from complex water matrices.

## Abstract 2

A magnetically recoverable Fe_3_O_4_@MIL-100(Fe)/CNT composite enables the simultaneous removal of polystyrene micro- and nanoplastics from different water matrices.

## Graphical abstract

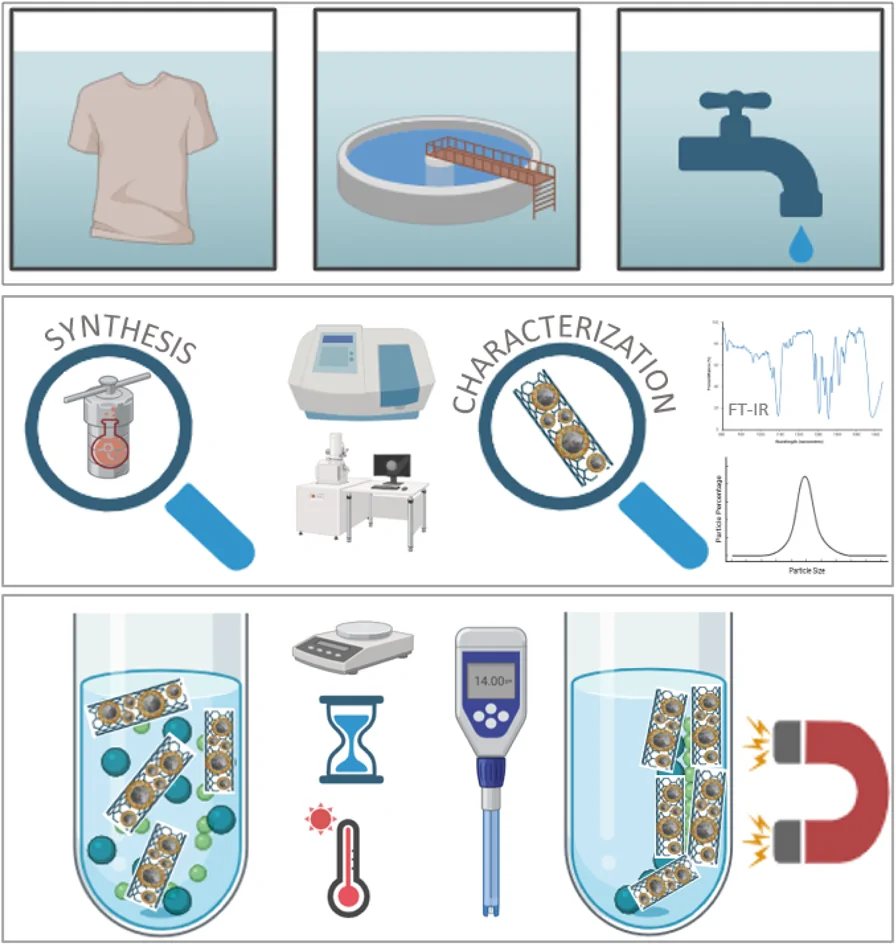

## 1 Introduction

Plastics are widely used in almost every aspect of modern life due to their advantages such as low cost, light weight, and high durability.^1^ However, the continued growth in plastic production has led to the persistent accumulation of plastic waste in the environment.^2,3^ Due to their poor biodegradability, plastic residues undergo mechanical abrasion, photooxidation, and biodegradation processes, transforming into microplastics (MPs, 5 mm–1 µm) and nanoplastics (NPs, <1 µm). These particles are now recognized as emerging contaminants because of their persistence, mobility and ability to interact with other pollutants in aquatic systems.^4–9^ More importantly, the high surface area and hydrophobic nature of MPs/NPs facilitate the adsorption of toxic compounds such as heavy metals, dyes, phthalates, and persistent organic pollutants, thereby increasing their transport throughout the food chain.^10^ These characteristics increase their environmental relevance and complicate their removal from water.

Current MP/NP removal technologies are primarily based on filtration, sedimentation, and coagulation. However, membrane filtration presents problems such as membrane fouling and increased operating costs,^11^ sedimentation shows lower effectiveness with small particles^12^ and coagulation generally requires the use of high doses of coagulants such as Al^3+^ salts.^13^ Therefore, in recent years, alternative approaches such as membrane-based processes, adsorption, advanced oxidation processes, artificial wetlands, electrocoagulation, and flotation have been intensively investigated.^14–28^ Among these methods, adsorption stands out due to its operational simplicity, economic feasibility, and suitability for regeneration.

Carbon-based adsorbents have shown promising results for MP/NP removal^29^ however the adsorption performance of conventional carbon-based materials can be limited in some cases. Carbon nanotubes (CNTs), especially multi-walled CNTs, are noteworthy adsorbent candidates due to their porous structure, hydrophobic character, high accessible surface area, and suitability for composite design.^30,31^ In particular, carboxylated MWCNTs offer suitable functional groups for the immobilization of metal–organic frameworks (MOFs) and it has been reported that the addition of MOFs improves the adsorption properties of these structures.^32^ However, the very fine particle size and agglomeration tendency of CNTs limit their practical use therefore composite strategies become necessary.^33–35^

MOFs are highly porous crystalline materials formed by the coordination of metal ions or clusters with organic ligands, and are attracting widespread interest due to their high surface areas, tunable pore structures, and functional versatility.^36–38^ MOFs are being extensively researched for applications in fields such as sensors, energy storage, pharmaceutical delivery, catalysis^39^ and water treatment.^39–46^ MOF-based materials have been shown to exhibit promising performance in the removal of heavy metals, dyes, organic microcontaminants, tetracyclines and recently MP/NPs.^47–56^ However, the separation and recovery of powdered MOFs from the post-treatment medium presents a significant practical limitation. In this context, magnetic adsorbents offer a significant advantage in terms of reuse and process optimization due to their rapid recovery from the solution under an external magnetic field.^57,58^ Zhang and Yu^59^ reported that the incorporation of Fe_3_O_4_ into a composite adsorbent enabled its rapid separation from water using an external magnetic field and facilitated its recovery for subsequent reuse.

In this study, the competitive adsorption of PS MPs and PS NPs from water was investigated at the interface of a magnetically recoverable hybrid mFMC. Unlike studies focusing on a single particle fraction, this work compares single and binary component systems to examine how particle size influences adsorption when MPs and NPs compete for the same hybrid interface. The effects of adsorbent dosage, initial particle concentration, contact time, pH and temperature were evaluated. Adsorption behavior was interpreted using zeta potential measurements, classical DLVO calculations, Raman spectroscopy, BET and SEM results, with particular emphasis on surface charge, hydrophobic interactions and π–π interactions. The performance of the hybrid was also examined in drinking water, urban wastewater treatment plant effluent and textile wastewater, together with five adsorption–desorption cycles. By linking competitive adsorption with interfacial characterization, this study provides a mechanistic perspective on the simultaneous capture of PS micro and nanoplastics by a magnetic MOF/CNT hybrid.

## 2 Materials and methods

### 2.1 Chemicals and reagents

Sodium hydroxide (NaOH) and anhydrous sodium acetate (NaAC) were obtained from Merck (Darmstadt, Germany). Iron(iii) chloride hexahydrate (FeCl_3_·6H_2_O) and benzene-1,3,5-tricarboxylic acid (H_3_-btc) were obtained from PerkinElmer (Turku, Finland). Ethylene glycol (EG), 2-morpholinoethanesulfonic acid monohydrate (MES), and ethanol were obtained from Sigma-Aldrich (Taufkirchen, Germany). *N*,*N*-Dimethylformamide (DMF) were obtained from Merck (Darmstadt, Germany). Fluorescent MP with a diameter of 1 µm and fluorescent NP with a diameter of 0.1 µm were obtained from Lab 261 (USA). Multi-walled carbon nanotubes (>99% purity) were obtained from Nanografi (Turkey).

### 2.2 Synthesis of mFMC composite

#### 2.2.1 Synthesis of Fe_3_O_4_ nanoparticles

**Fig. 1:**
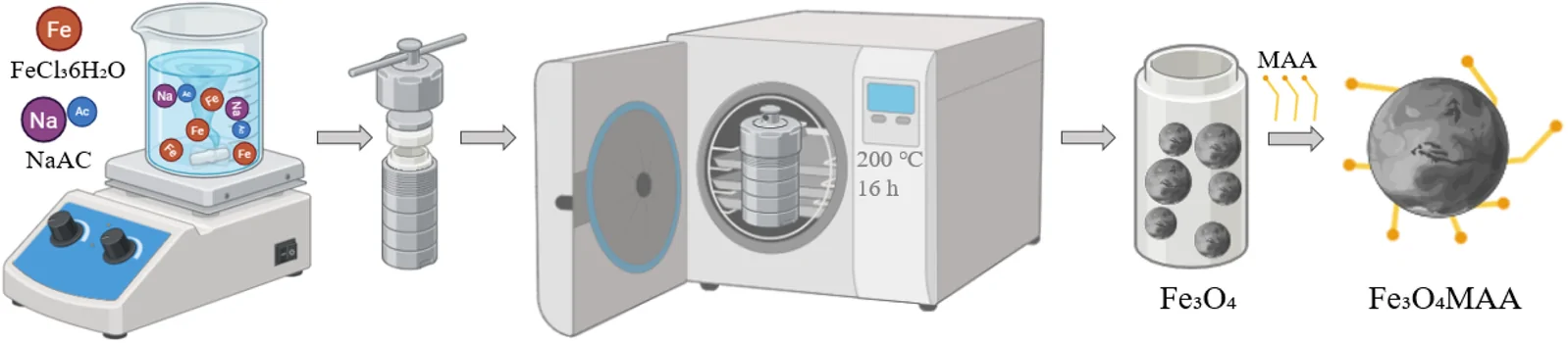
Synthesis scheme for surface functionalization of Fe_3_O_4_ nanoparticles with MAA.

Fe_3_O_4_ nanoparticles were prepared by a solvothermal route. FeCl_3_·6H_2_O (1.25 g) was first dissolved in 80 mL of ethylene glycol and anhydrous sodium acetate (3.2 g) was subsequently added. After 30 min of stirring, the mixture was transferred to a Teflon-lined stainless-steel autoclave and maintained at 200 °C for 16 h. Once the autoclave had cooled to room temperature, the magnetic particles were recovered with an external magnet, washed three times with ethanol and dried at 60 °C. For surface functionalization, 342 mg of Fe_3_O_4_ was dispersed in 80 mL of ethanol containing 0.58 mM mercaptoacetic acid. The sealed suspension was shaken overnight at room temperature under nitrogen. The resulting Fe_3_O_4_-MAA particles were magnetically collected, washed repeatedly with ethanol to remove residual reagent and dried overnight at 60 °C. The synthesis scheme for the surface functionalization of Fe_3_O_4_ nanoparticles with MAA is shown in Fig. 1.

#### 2.2.2 Preparation of Fe_3_O_4_@MOF core–shell composite structures

**Fig. 2:**
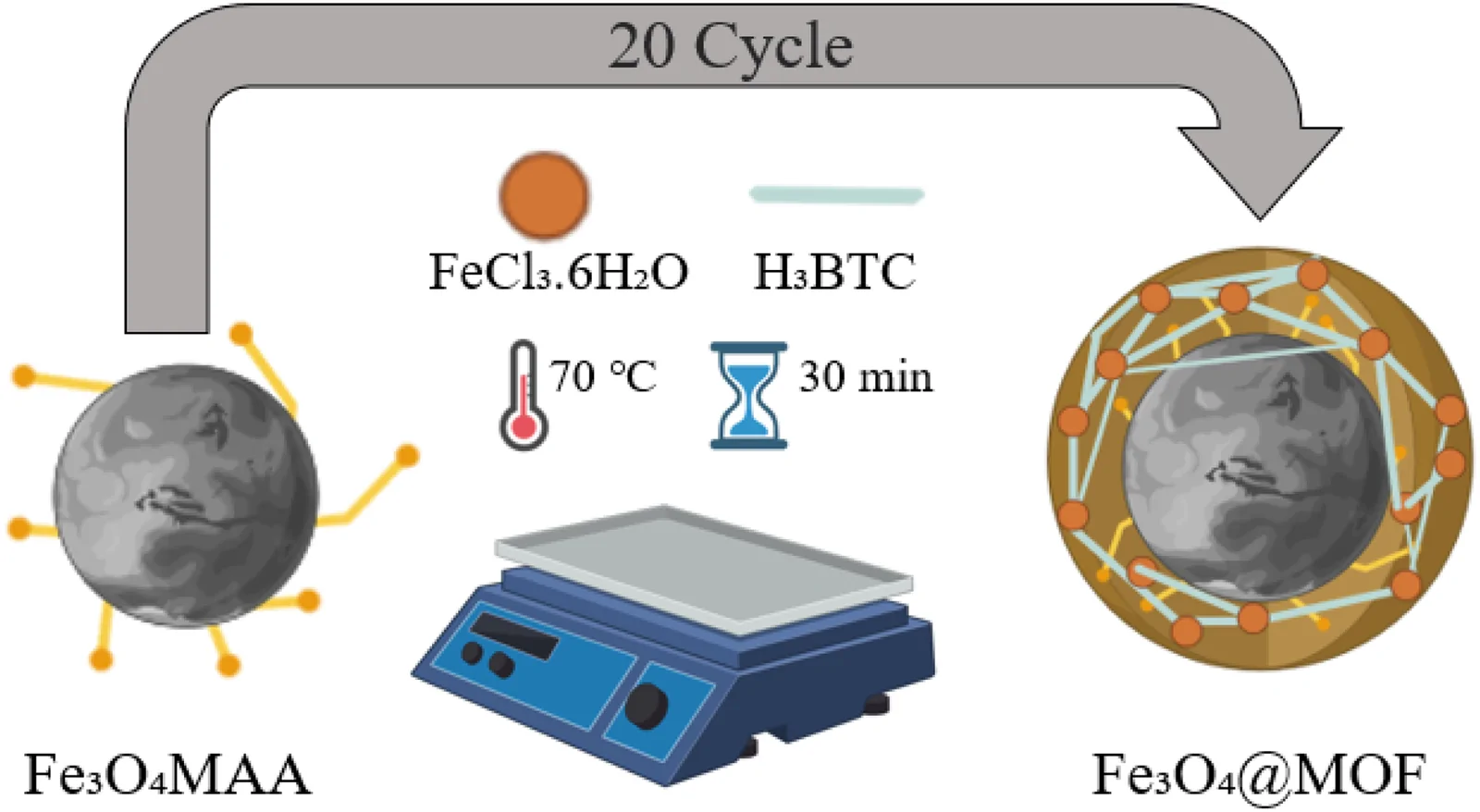
Schematic illustration of the synthesis process of the Fe_3_O_4_@MOF structure.

A step-by-step assembly method was used to synthesize porous MOF (MIL-100(Fe)) particles, and this was carried out in two stages. In the first stage, 100 mg of magnetic particles modified with MAA (Fe_3_O_4_MAA) were taken and added to a 5 mL ethanol solution containing 10 mM FeCl_3_·6H_2_O. After gently stirring for approximately 1 minute, it was left to stand still for 15 minutes. The magnetically collected particles were washed three times with ethanol. In the second stage, the collected magnetic particles were added to a mixture containing 10 mM H_3_btc prepared in 5 mL ethanol. It was kept in a shaking water bath at 70 °C for half an hour. The magnetically collected particles were washed three times with ethanol. These two successive stages constituted one deposition cycle. The cycle was repeated 20 times. After the final cycle, the particles were washed three times with ethanol and dried at 150 °C for 12 h. Fig. 2 shows the synthesis of Fe_3_O_4_@MOF.

#### 2.2.3 Preparation of mFMC composite

**Fig. 3:**
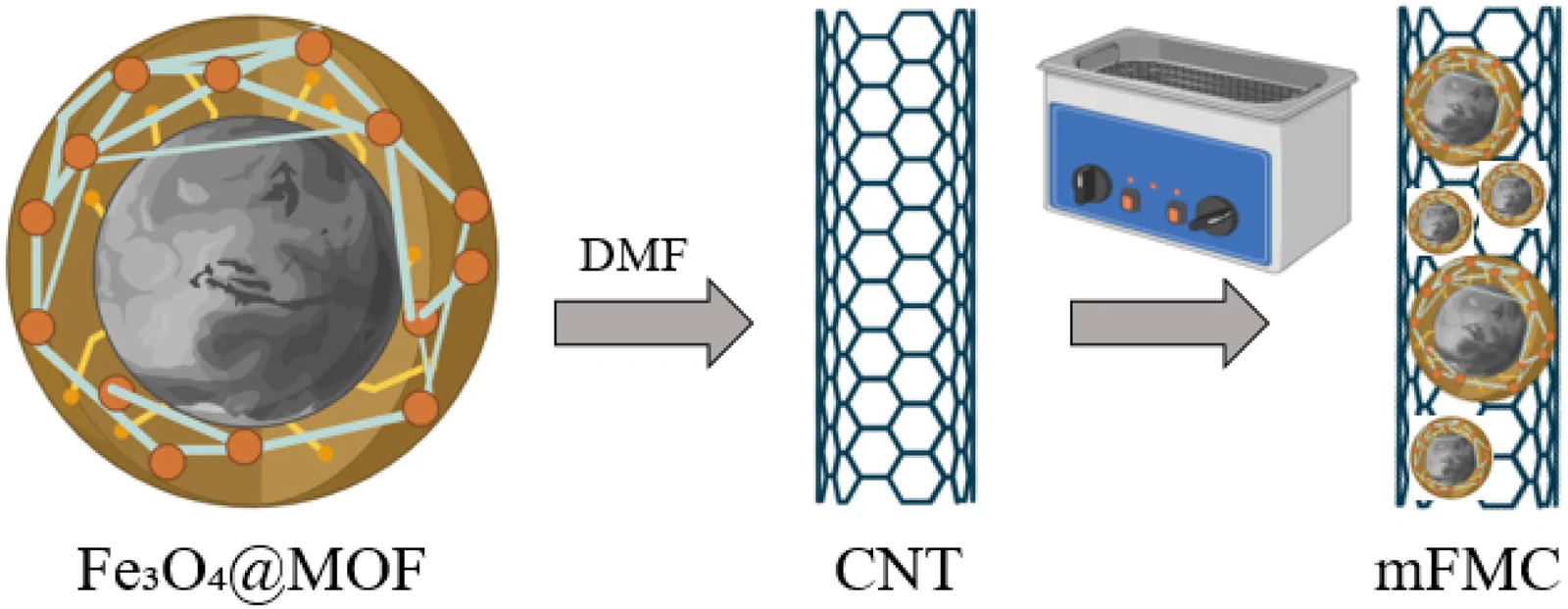
Schematic representation of a mFMC composite structure.

mFMC composites were prepared using different CNT : Fe_3_O_4_@MIL-100(Fe) mass ratios. Fe_3_O_4_@MIL-100(Fe) particles were added to CNT suspensions prepared in DMF at CNT : Fe_3_O_4_@MIL-100(Fe) ratios of 1 : 1, 1 : 2 and 2 : 1 (w/w). The mixtures were sonicated in an ultrasonic bath for 30 min to improve dispersion and promote contact between the components. The resulting suspensions were then centrifuged and washed several times with ethanol and distilled water. After washing, the composites were dried in an oven at 60 °C. Based on its higher adsorption performance, the composite prepared at a 1 : 1 CNT : Fe_3_O_4_@MIL-100(Fe) mass ratio was selected for subsequent characterization and adsorption experiments. Schematic representation of a mFMC composite structure is shown in Fig. 3.

### 2.3 Characterization of mFMC composite

The chemical, structural, morphological, magnetic and surface properties of the mFMC composite were characterized using comprehensive analytical techniques. The functional groups of the composite were verified using Fourier transform infrared spectroscopy (FT-IR, PerkinElmer Spectrum 400) over the range of 4000–400 cm^−1^ at a resolution of 4 cm^−1^ using 32 scans. The morphological structure, the distribution of MOF crystals on the CNT surface and the elemental composition were examined using scanning electron microscopy coupled with energy dispersive X-ray spectroscopy (SEM/EDS, FEI Quanta 400F). Before imaging, the samples were coated with a thin gold layer and examined under high vacuum at an accelerating voltage of 15 kV. Crystal phase analysis was performed over a 2*θ* range of 5–50° using X-ray diffraction (XRD, Rigaku MiniFlex). Thermal resistance and degradation behavior were investigated between 25 and 800 °C using thermogravimetric analysis (TGA, Hitachi STA 200) under a nitrogen atmosphere at a heating rate of 10 °C min^−1^. Magnetic properties were measured using a vibrating sample magnetometer (VSM, Dexing Magnet VSM 550). The structural integrity and bonding characteristics of the CNT and MIL-100(Fe) components were also examined using Raman spectroscopy (Renishaw inVia) with a 785 nm excitation laser over the range of 50–4000 cm^−1^. Specific surface area and pore-size distribution were determined using a Micromeritics BET surface area analyzer based on N_2_ adsorption–desorption isotherms. Surface charge and colloidal stability of the composite were measured using a zeta potential analyzer (Malvern Zetasizer Nano ZS90). The samples were dispersed in deionized water and measured at 25 °C in triplicate. The fluorescence intensities of the PS micro and nanoplastic suspensions were recorded using a fluorescence spectrophotometer (PerkinElmer FL6500) at excitation/emission wavelengths of 460/500 nm for PS NPs and 530/582 nm for PS MPs.

### 2.4 Batch adsorption experiments

Fluorescent polystyrene microplastics (PS MPs, 1 µm) and nanoplastics (PS NPs, 0.1 µm) were used as model plastic particles in the adsorption experiments. For the binary adsorption experiments, 4 mL of the PS MP suspension and 4 mL of the PS NP suspension were mixed at an equal volume ratio, giving a total reaction volume of 8 mL. Each particle type was present at an initial concentration of 50 µg mL^−1^, resulting in a total PS MP/NP concentration of 100 µg mL^−1^.

Standard calibration curves were prepared for PS MPs and PS NPs by measuring their fluorescence intensities at different concentrations. The calibration curves are given in the SI as Fig. S1(a and b). The fluorescence signals were recorded at excitation/emission wavelengths of 530/582 nm for PS MPs and 460/500 nm for PS NPs. Spectral cross-talk was tested at both fluorescence channels and was negligible. After adsorption, the mFMC hybrid was removed from the suspension using an external magnet and the fluorescence intensity of the supernatant was measured. The residual PS MP and PS NP concentrations were then calculated separately using the corresponding calibration equations.

Batch adsorption experiments were performed to examine the effects of adsorbent dosage, initial PS MP/NP concentration, contact time, pH and temperature. The adsorbent dosage was varied from 1.0 to 3.5 mg and the initial PS MP/NP concentration was studied in the range of 70–110 µg mL^−1^. The mixtures were agitated in an orbital shaker at 180 rpm for contact times of 15 min, 30 min, 1 h, 3 h, 6 h, 12 h, 24 h and 36 h. At the end of each experiment, the adsorbent was magnetically separated, and the remaining PS MP and PS NP concentrations in the supernatant were determined by fluorescence spectrophotometry.

To evaluate competitive adsorption, PS MPs and PS NPs were tested both separately and together. In the single-component experiments, PS MPs or PS NPs were studied separately at an initial concentration of 100 µg mL^−1^. In the binary system, both particle types were present at 50 µg mL^−1^, resulting in a total PS concentration of 100 µg mL^−1^. This comparison made it possible to observe how the presence of one particle type affected the adsorption behavior of the other.

The effect of solution pH was investigated between pH 2 and 11 by adjusting the initial pH with 0.1 M HCl or 0.1 M NaOH. Temperature-dependent adsorption experiments were conducted at 298, 308 and 318 K. Unless otherwise stated, the experiments were conducted using 2.5 mg of mFMC, a total reaction volume of 8 mL, an initial total PS MP/NP concentration of 100 µg mL^−1^, a shaking rate of 180 rpm and a contact time of 360 min. After magnetic separation, the remaining PS MP and PS NP concentrations were determined as described above.

Removal efficiency and adsorption capacity (*Q*_e_) were calculated according to eqn (S1) and (S2) in the SI. All adsorption experiments were performed in triplicate, and the results were reported as mean ± standard deviation.

### 2.5 DLVO interaction-energy calculations

Classical DLVO calculations were performed to semi-quantitatively evaluate the pH-dependent interactions of PS MPs and PS NPs with the mFMC surface. The total interaction energy, *V*_T_, was expressed as the sum of the electrostatic double-layer and van der Waals interaction energies:

1 *V*_T_(h) = *V*_EDL_(h) + *V*_vdW_(h)

A constant-potential Hogg–Healy–Fuerstenau sphere-plate model was used with the spherical PS particle approaching a locally planar region of the heterogeneous mFMC surface. Constant potential electrolyte interaction models provide a practical framework for evaluating interactions between surfaces carrying dissimilar potentials.^60^ Experimentally measured zeta potentials were used as approximations of the interacting surface potentials, recognizing that zeta potential corresponds to the potential at the slipping plane.^61,62^ Nominal radius of 500 and 50 nm were used for PS MPs and PS NPs, respectively.

The ionic environment was represented by the directly measured conductivities of the equal-volume MP/NP binary suspensions at each pH, which were converted to KCl-equivalent ionic strengths.^63^ Since KCl-based conversion does not fully account for the higher ionic mobilities of H^+^ and OH^−^ introduced during pH adjustment, the resulting values – obtained using KCl as the standard conductivity reference electrolyte^63^ – represent reasonable approximations. A representative effective Hamaker constant of 1.0 × 10^−20^ J was used for the PS-water-mFMC system and the robustness of the calculated interaction energy barriers was evaluated over the *A*_132_ range of 0.5–3.0 × 10^−20^ J.^64^ Interaction energy profiles were calculated over separation distances of 0.5–100 nm and normalized by *k*_B_*T*. The complete equations, calculation parameters, conductivity conversion and Hamaker-constant sensitivity analysis are provided in SI.

### 2.6 Adsorption kinetics of mFMC composite on PS MP/NP

The adsorption kinetics of NP and MP were evaluated using the pseudo-first-order, pseudo-second-order and intraparticle diffusion models. The corresponding equations are provided in the SI.

### 2.7 Adsorption isotherms of mFMC composite on PS MP/NP surfaces

Classical density functional theory (cDFT) provides a rigorous molecular-level framework for describing polymer adsorption and the distribution of polymer segments near solid interfaces.^65,66^ The present study, however, focuses on the experimental equilibrium behavior of micro- and nanoscale PS particles on a heterogeneous composite surface. Therefore, the Langmuir and Freundlich isotherm models were used to correlate the experimental data and enable direct comparison with previously reported MP/NP adsorption studies. The corresponding equations for these adsorption models are presented in the SI.

### 2.8 Reusability of mFMC composite

Recovery after the adsorption process was achieved by magnetically separating the mFMC adsorbent from water. 1 mL of ethanol was added to the magnetically collected mFMC and subjected to an ultrasonic bath for 30 minutes. It was redistributed in a fresh NP and MP mixture solution to initiate the next adsorption cycle. The process was repeated for five consecutive adsorption–desorption cycles.

### 2.9 Adsorption in real samples

To evaluate the performance of the mFMC composite under environmentally relevant conditions, three real water matrices were tested: drinking water, wastewater treatment plant effluent, and textile wastewater. Drinking water was obtained from the outlet of the municipality's urban drinking water treatment plant, and wastewater treatment plant effluent was obtained from the final sedimentation outlet of the same municipality's urban wastewater treatment plant. Textile wastewater was collected from the dyeing process outlet of a textile plant in the region. After collection, the samples were passed through a 0.45 µm membrane filter, and adsorption experiments were performed immediately. The total PS MP/NP concentration in each real water sample was adjusted to 100 µg mL^−1^. Then, 2.5 mg of the mFMC composite was added, and the mixtures were stirred in a shaker incubator for the specified contact time. After adsorption, the composite was magnetically separated from the suspension, and the residual MP/NP concentrations were measured using a fluorescence spectrophotometer. Spike-recovery tests were performed in triplicate, yielding recoveries of 99.87 ± 4.01%, 93.17 ± 3.07% and 92.20 ± 2.72% for tap water, textile wastewater and wastewater treatment plant effluent, respectively.

## 3 Results and discussions

### 3.1 Characterization of mFMC composite

**Fig. 4:**
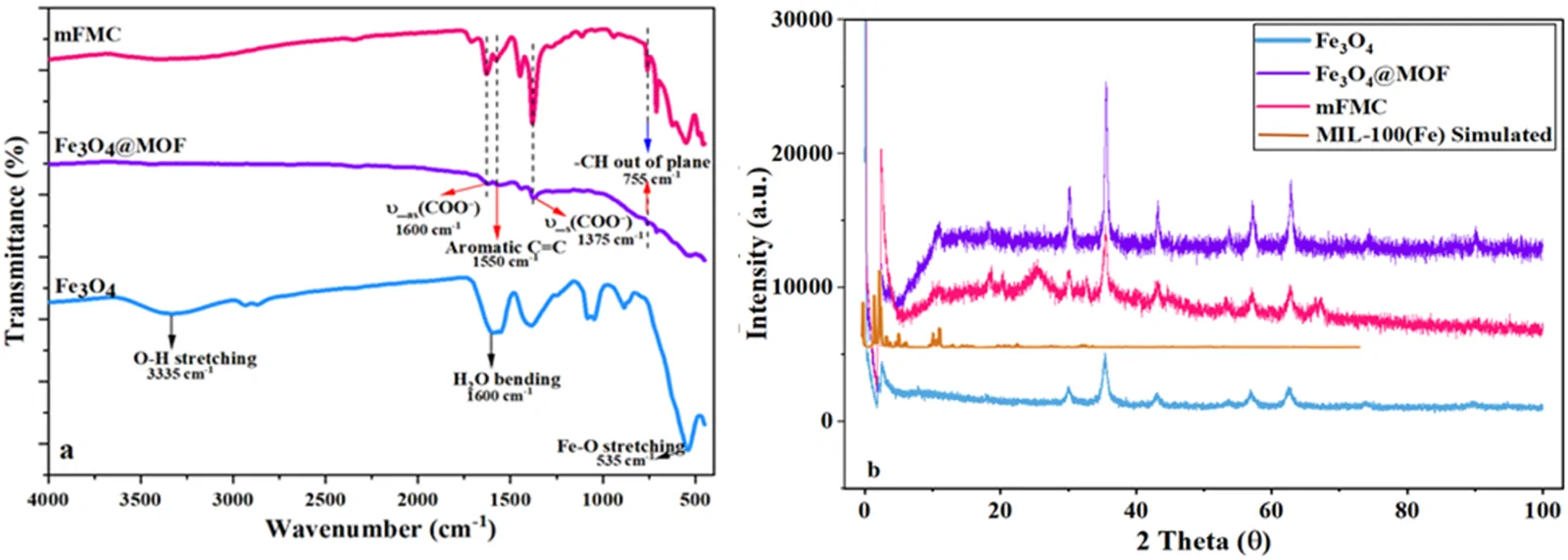
ATR-IR spectra of Fe_3_O_4_, Fe_3_O_4_@MOF and mFMC samples (a) XRD patterns of Fe_3_O_4_, Fe_3_O_4_@MOF, and mFMC samples (b).

The ATR-IR spectra of Fe_3_O_4_, Fe_3_O_4_@MOF and mFMC samples are presented in Fig. 4(a). In the spectrum of pure Fe_3_O_4_ nanoparticles, a distinct and sharp band observed at approximately 535 cm^−1^ is attributed to the Fe–O stretching vibration, confirming the successful formation of the magnetite structure.^67,68^ In addition, the bands observed around 3335 cm^−1^ and 1600 cm^−1^ are assigned to the O–H stretching vibrations and the bending vibrations of adsorbed water (H_2_O) molecules, respectively.^69^ In the spectrum of the Fe_3_O_4_@MOF structure, the band appearing around 1620 cm^−1^ is attributed to the asymmetric stretching vibration of carboxylate (COO^−^) groups. In contrast, the band observed at approximately 1375 cm^−1^ is associated with the symmetric stretching vibration. These bands indicate the coordination interaction between metal ions and organic ligands, confirming the successful formation of the MOF structure.^70^ Furthermore, the band around 1550 cm^−1^ can be attributed to aromatic ring vibrations. The band observed at approximately 1040 cm^−1^ is assigned to C–O/C–C stretching vibrations, while the band around 755 cm^−1^ corresponds to the out-of-plane bending vibrations of aromatic –CH groups.^71,72^ The observed peaks are in good agreement with those previously reported for pure MIL-100(Fe). In particular, the characteristic bands reported in the ranges of 1620–1626, 1543–1550, 1371–1396 and 750–760 cm^−1^ are consistent with the bands observed at 1620, 1550, 1375 and 755 cm^−1^, respectively, for the Fe_3_O_4_@MOF sample.^73,74^ This agreement supports the successful creation of the MIL-100(Fe) framework on the Fe_3_O_4_ core. In the mFMC hybrid structure, the characteristic bands of the MOF are generally preserved, indicating that the incorporation of CNTs does not disrupt the fundamental crystal and chemical framework of the MOF structure.^75^ However, a noticeable increase in the intensity of the band in the range of approximately 1580–1620 cm^−1^ is observed. This enhancement is attributed to the C=C stretching vibrations of graphitic carbon in CNTs, confirming their successful integration into the MOF structure.^76,77^ In addition, the absence of new characteristic bands and the lack of significant peak shifts suggest that the interaction between CNTs and the MOF is predominantly physical, while the structural integrity of the system is maintained.^76^ Overall, the FTIR results clearly confirm the successful formation of the mFMC hybrid structure and demonstrate that the incorporation of CNTs occurs without compromising the structural stability of the system.

Fig. 4(b) shows the X-ray diffraction (XRD) patterns of pristine Fe_3_O_4_, Fe_3_O_4_@MOF and mFMC samples. In the XRD pattern of Fe_3_O_4_ nanoparticles, well-defined diffraction peaks are observed at 2*θ* values of 30.09°, 35.35°, 43.06°, 53.57°, 56.88°, 62.52° and 73.88°, which correspond to the (220), (311), (400), (422), (511), (440), and (533) crystallographic planes, respectively.^78–80^ These diffraction peaks are in good agreement with the standard cubic spinel structure of Fe_3_O_4_ (JCPDS card no. 01-079-0417), confirming the successful synthesis of magnetite nanoparticles.^81^ In the XRD pattern of Fe_3_O_4_@MOF, the characteristic diffraction peaks of Fe_3_O_4_ are still clearly observed, indicating that the crystalline structure of Fe_3_O_4_ is preserved after MOF formation. In addition, a diffraction peak appearing at around 2*θ* ≈ 11° is consistent with the characteristic reflection of the MIL-100(Fe) structure reported in the literature.^82,83^ The emergence of this peak confirms the successful growth of the MOF layer on the Fe_3_O_4_ surface. For comparison, the simulated XRD pattern of MIL-100(Fe) is also included in Fig. 4(b). The characteristic peaks of Fe_3_O_4_@MOF are consistent with the simulated MIL-100(Fe) pattern, supporting the formation of the MIL-100(Fe) framework on the Fe_3_O_4_ surface.^84^ Upon incorporation of CNTs into the Fe_3_O_4_@MOF structure, a broad diffraction peak is observed at approximately 2*θ* ≈ 25.5°, which corresponds to the (002) plane of graphitic carbon. This broad peak is attributed to the characteristic reflection of CNTs and is consistent with the standard graphite structure (JCPDS No. 01-0646).^85,86^ The broad nature of this peak indicates the partially amorphous and disordered structure of CNTs. Moreover, the preservation of the main diffraction peaks of Fe_3_O_4_ and MOF, along with the appearance of the CNT-related peak, clearly demonstrates the successful formation of the mFMC hybrid structure. These results indicate that the stepwise synthesis strategy leads to the formation of a well-defined hybrid system without disrupting the crystalline integrity of the individual components.

**Fig. 5:**
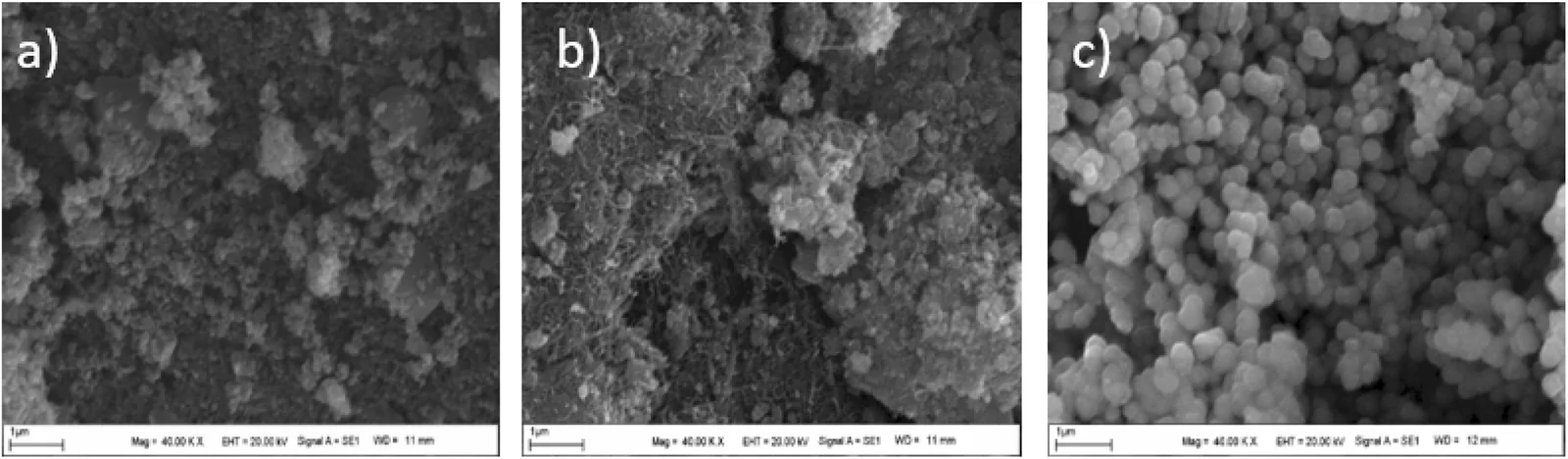
SEM images of (a) Fe_3_O_4_, (b) Fe_3_O_4_@MOF and (c) mFMC samples.

The surface morphologies of Fe_3_O_4_, Fe_3_O_4_@MOF and mFMC samples were investigated by scanning electron microscopy (SEM), and the corresponding images are presented in Fig. 5(a–c). The SEM image of pristine Fe_3_O_4_ nanoparticles (Fig. 5(a)) reveals that the particles exhibit predominantly spherical and semi-spherical morphologies with a noticeable tendency to agglomerate.^87^ This aggregation behavior can be attributed to strong magnetic dipole–dipole interactions and the high surface energy of Fe_3_O_4_ nanoparticles.^88,89^ The particles are observed to be in the nano to submicron size range, with relatively rough and irregular surfaces.^90,91^ In the case of the Fe_3_O_4_@MOF sample (Fig. 5(b)), a noticeable change in surface morphology is observed compared to pristine Fe_3_O_4_. The particle surfaces become more irregular and rough, indicating the formation of a MOF layer on the Fe_3_O_4_ surface.^91^ The appearance of a more heterogeneous and textured morphology suggests the successful growth of the MOF structure and an increase in surface complexity.^92,93^ For the mFMC sample (Fig. 5(c)) a distinct morphological transformation is observed, where a fibrous network structure associated with CNTs is clearly visible.^93^ In addition, bead-like structures distributed along the CNT network are observed. This bead-on-string-like morphology indicates that Fe_3_O_4_@MOF particles are effectively anchored onto the CNT surface, forming an interconnected hybrid architecture.^94,95^ The presence of CNTs not only introduces a network-like structure but also acts as a bridging component, enhancing the structural integrity of the system.^89^

Overall, the SEM results clearly demonstrate a progressive morphological evolution from Fe_3_O_4_ to Fe_3_O_4_@MOF and finally to the mFMC hybrid structure. These findings confirm that the stepwise synthesis strategy enables the successful integration of each component into a well-defined and interconnected hybrid system.

**Fig. 6:**
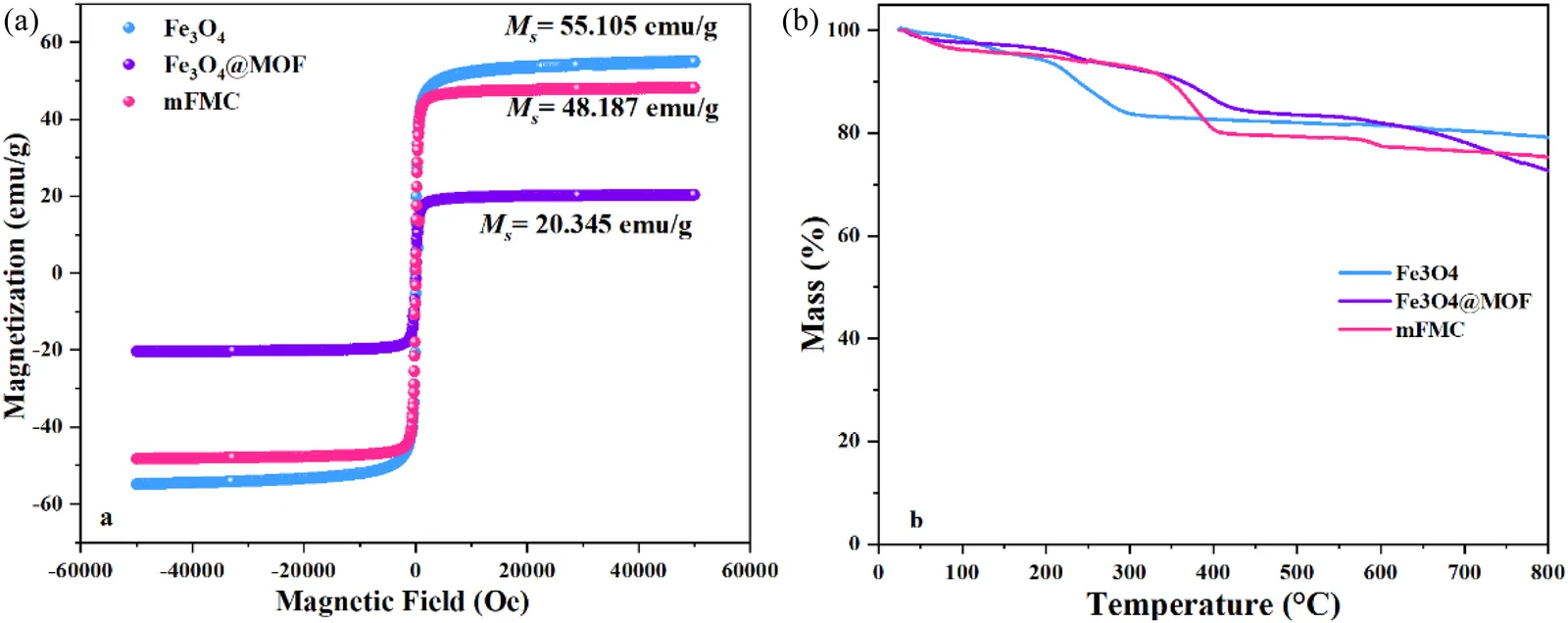
Magnetization curves of Fe_3_O_4_, Fe_3_O_4_@MOF and mFMC samples (a), TGA curves for Fe_3_O_4_, Fe_3_O_4_@MOF and mFMC samples (b).

The magnetic properties of pristine Fe_3_O_4_, Fe_3_O_4_@MOF and mFMC samples were investigated using a Vibrating Sample Magnetometer (VSM) at 300 K. The magnetization curves as a function of the applied magnetic field are presented in Fig. 6(a). The saturation magnetization (*M*_s_) value of the synthesized Fe_3_O_4_ nanoparticles was determined to be 55.105 emu g^−1^, which is consistent with previously reported values for magnetite nanoparticles.^78,96^ After modification with the MOF structure, the *M*_s_ value significantly decreased to 20.345 emu g^−1^. This reduction can be attributed to the non-magnetic nature of the MOF shell, which partially isolates the magnetic Fe_3_O_4_ core and reduces the overall magnetic moment per unit mass. Similar reductions in *M*_s_ values for Fe_3_O_4_@MOF composites compared to bare Fe_3_O_4_ have been widely reported and are associated with surface coating effects and magnetic dilution.^97^ In addition, the coating layer weakens magnetic dipole–dipole interactions, further decreasing the overall magnetic response.^98^

Following CNT incorporation, the *M*_s_ value increased again to 48.187 emu g^−1^. This enhancement is attributed to the conductive and network-like structure of CNTs, which improves interparticle interactions and facilitates charge/electron transfer within the composite. Previous studies have demonstrated that CNT incorporation into MOF-based composites enhances structural integrity and strengthens interfacial interactions, thereby improving overall performance.^99,100^ Furthermore, CNT-containing magnetic hybrid systems can generate synergistic effects between magnetic and dielectric properties, contributing to enhanced functional behavior.^101^ Consequently, the contribution of the magnetic core becomes more pronounced within the hybrid structure. The relatively high saturation magnetization values indicate that the synthesized composites can be efficiently separated under an external magnetic field, suggesting their strong potential for magnetic separation applications.^102,103^

The thermal behaviors of pristine Fe_3_O_4_, Fe_3_O_4_@MOF and mFMC samples were systematically investigated by thermogravimetric analysis (TGA) and the corresponding results are presented in Fig. 6(b). A slight weight loss observed in all samples within the temperature range of 0–150 °C is attributed to the removal of adsorbed water molecules and physically bound moisture from the surface. Such initial weight losses are commonly reported in the literature for Fe_3_O_4_ and its derivative composites.^104^ The TGA curve of pristine Fe_3_O_4_ nanoparticles exhibits minimal weight loss over a broad temperature range, demonstrating their excellent thermal stability. This behavior can be attributed to the robust inorganic structure of magnetite and its high residual mass at elevated temperatures.^87,105^ In the Fe_3_O_4_@MOF sample, a significant weight loss occurs within the temperature range of 200–400 °C, which can be attributed to the thermal decomposition of the organic ligands constituting the MOF framework. In MOF-based hybrid structures, degradation in this region is typically associated with the decomposition of organic linkers and is widely regarded as an indication of the successful growth of the MOF layer on the Fe_3_O_4_ core.^106,107^ The mFMC sample also exhibits weight loss associated with the presence of organic components; however, a more controlled degradation behavior is observed compared to the Fe_3_O_4_@MOF sample. This improved thermal behavior can be attributed to the network-like structure of CNTs, which contributes to structural stabilization and shifts the decomposition process to higher temperatures.^108,109^ In the high-temperature region (600–800 °C), the residual mass analysis indicates that pristine Fe_3_O_4_ exhibits the highest residue, while the Fe_3_O_4_@MOF sample shows a lower residual mass due to the presence of thermally decomposable organic components. The mFMC sample displays an intermediate behavior between these two systems. These results further confirm the direct correlation between the inorganic phase fraction and the final residual mass.^110^ These findings demonstrate that surface modification significantly influences the thermal stability and that the hybrid structure exhibits a controlled thermal degradation behavior. Moreover, the interactions between nanoparticles, the matrix, and CNTs play a critical role in delaying the decomposition process and improving thermal resistance, in good agreement with previous reports in the literature.^111^

### 3.2 Optimization of MP/NP adsorption experiments

#### 3.2.1 Effect of adsorbent dosage and initial concentration

**Fig. 7:**
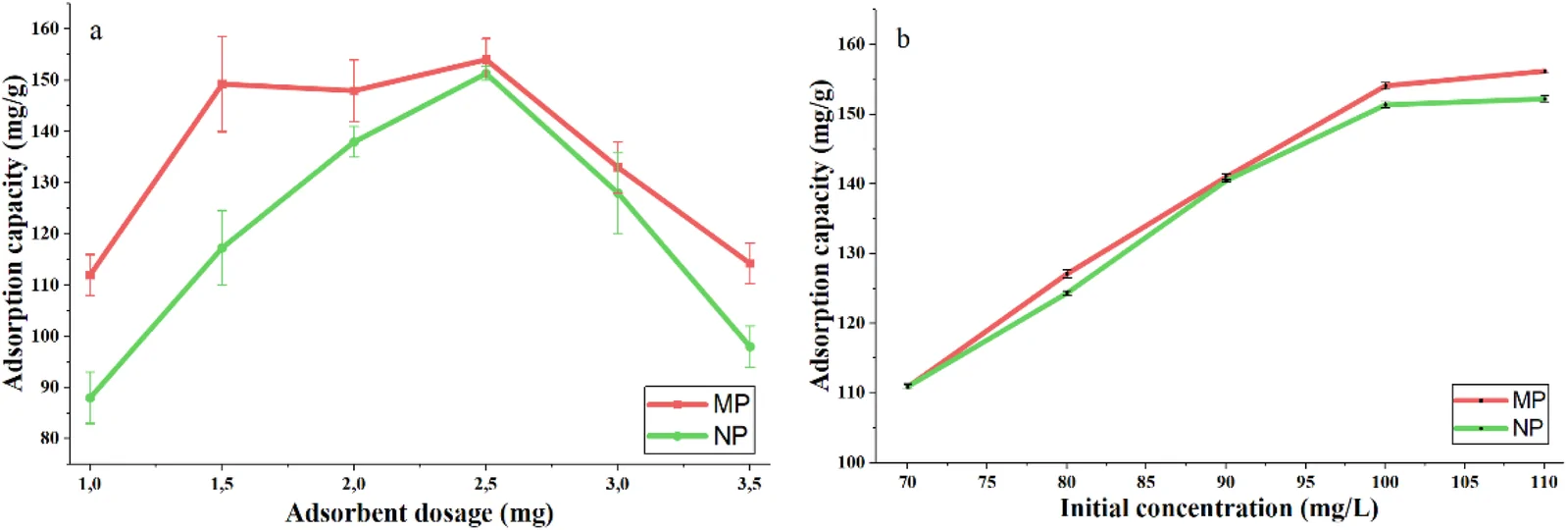
The effect of mFMC dosage on the adsorption capacity of PS MP/NPs (pH: 7, temperature: 25 °C, total MP/NP concentration: 100 µg mL^−1^) (a), the effect of initial MP/NP concentration on adsorption capacity (pH: 7, temperature: 25 °C, mFMC dosage: 2.5 mg) (b).

Fig. 7(a) shows the effects of mFMC amount on the removal efficiency and adsorption amount of PS MPNPs. As the adsorbent amount increased from 1 mg to 2.5 mg, the total *Q*_e_ value increased from 200 mg g^−1^ to 305.44 mg g^−1^, and this increase is associated with the increase in the number of accessible active binding sites for PS MP/NP particles with increasing dosage.^112^ However, at dosages above 2.5 mg, *Q*_e_ values decreased significantly; at dosages of 3 mg and 3.5 mg, the total capacity dropped to 266 and 228.5 mg g^−1^, respectively. Based on these findings, the optimum adsorbent dosage was determined to be 2.5 mg, and all subsequent experiments were performed at this dosage. Additionally, as the PS MP/NP concentration was increased from 70 µg mL^−1^ to 100 µg mL^−1^, the *Q*_e_ values for MP and NP increased from 110.88 to 154.08 mg g^−1^ and from 110.88 to 151.36 mg g^−1^, respectively (Fig. 7(b)). Increasing the concentration to 110 µg mL^−1^ resulted in *Q*_e_ values remaining almost the same, but the MP/NP removal efficiency dropping below 90%. This was interpreted as an indication that the active sites on the adsorbent surface had reached saturation. Based on these findings, the initial concentration of 100 µg mL^−1^, where the adsorbent capacity is used most efficiently, was determined as the optimum condition for subsequent experiments.

In the literature, magnetic adsorbents have been shown to be an effective method for the removal of PS nanoplastics. Zhao *et al.*^113^ robtained an adsorption capacity of 82.8–89.9 mg g^−1^ for PS nanoplastics using Fe-modified magnetic fly ash, while Xing *et al.*^114^ reported a maximum adsorption capacity of 54.07 mg g^−1^ with CTAB and magnetic rice straw biochar modification. Among MOF-based materials, Wan *et al.*^115^ reported 34.5 mg g^−1^ for PS microplastics with ZIF-67, Peng *et al.*^116^ reported 94 mg g^−1^ with ZIF-67/CuCo-LDH composite, Yakout and Zainy^117^ reported 97.69 mg g^−1^ with chitosan/graphitic carbon nitride/ZIF-67 composite, and Zhou *et al.*^56^ found an adsorption capacity of 65.5 mg g^−1^ for PS nanoplastics with UiO-66/melamine foam composite. Compared with these results, mFMC exhibits high adsorption capacity for both PS microplastics and PS nanoplastics.

#### 3.2.2 Size dependent competitive adsorption between PS MPs and PS NPs

**Fig. 8:**
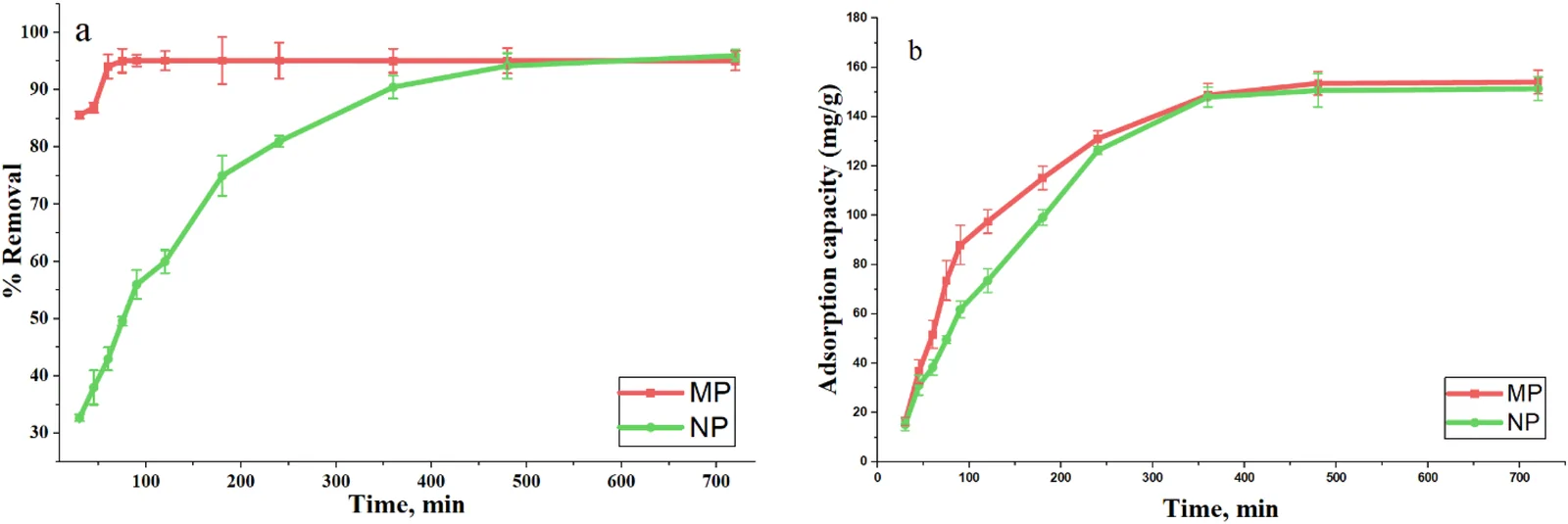
Effect of contact time on (a) PS-MP and PS-NP adsorption in a single component systems (pH: 7, temperature: 25 °C, initial concentration: 100 µg mL^−1^, mFMC dosage 2.5 mg) and (b) competitive adsorption of PS-MP and PS-NP in a binary system (pH: 7, temperature: 25 °C, total MP/NP concentration: 100 µg mL^−1^, mFMC dosage 2.5 mg).

To determine the equilibrium time of PS MP/NP adsorption onto the magnetic mFMC composite, the effect of contact time was investigated in both single and binary component systems. As shown in Fig. 8(a), PS MP adsorption in the single component system occurred rapidly and reached approximately 95% removal within 60 min. This rapid uptake can be attributed to the immediate availability of adsorption sites on the external surface of the hybrid.^115,118^ In contrast, PS NP adsorption occurred more slowly and approached equilibrium after approximately 480 min. This behavior may be related to the transport of the smaller particles toward the hybrid surface. Owing to their smaller size and higher mobility, PS NPs may access the external surface, interparticle voids and CNT-associated spaces more readily than PS MPs.^119^ A different adsorption pattern was observed when PS MPs and PS NPs were present together, as shown in Fig. 8(b). In the binary system, PS MP removal remained at approximately 38% during the first 120 min and increased to above 90% after 360 min. Given the smaller size and higher diffusivity of PS NPs relative to PS MPs, their more rapid access to the mFMC interface offers one plausible explanation for the delayed initial uptake of PS MPs observed in the binary system.^120^ However, further analyses, such as site-resolved spectroscopic or microscopic techniques, would be required to confirm preferential occupation of the adsorption interface by PS NPs.

Despite the slower initial adsorption of PS MPs, high removal efficiencies were achieved for both particle fractions at longer contact times. This behavior suggests that competition mainly influenced the adsorption rate rather than preventing the eventual capture of either fraction. The comparison between the single and binary component systems therefore highlights the role of particle size in controlling access to the mFMC interface and shows that adsorption behavior in mixed plastic suspensions cannot be predicted solely from single component experiments.

#### 3.2.3 pH dependent interfacial interactions and surface charge effects

**Fig. 9:**
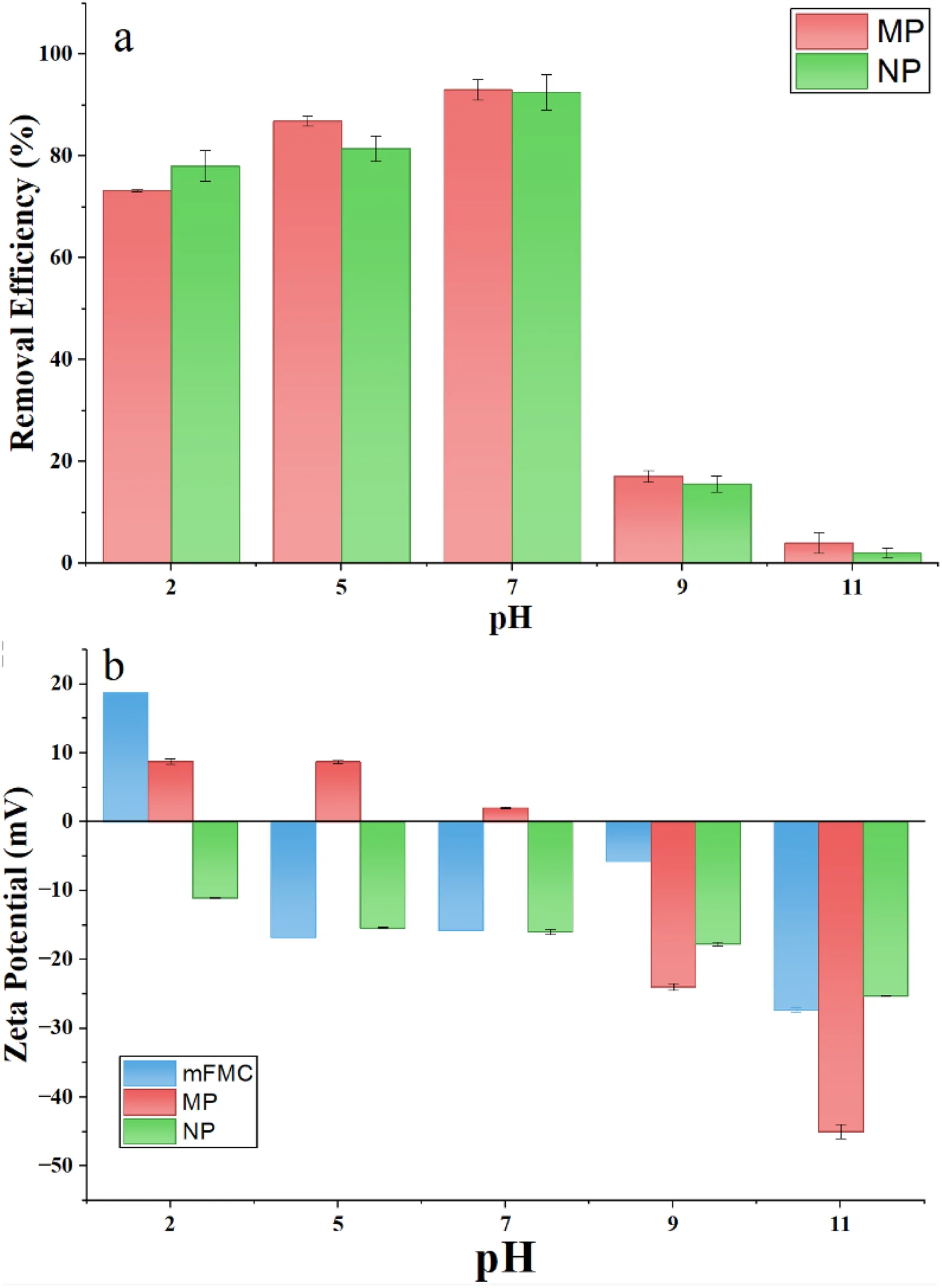
Effect of pH on the simultaneous removal of PS MP/NP particles by the mFMC composite (pH: 7, temperature: 25 °C, total MP/NP concentration: 100 µg mL^−1^, mFMC dosage 2.5 mg) (a) and zeta potential (pH: 7, temperature: 25 °C, total MP/NP concentration: 100 µg mL^−1^, mFMC dosage 2.5 mg) (b).

Solution pH is a key parameter in adsorption processes because it influences the surface charge of the adsorbent, the ionization state of surface functional groups and the interactions between the adsorbent and the target particles. Therefore, the effect of pH on the simultaneous adsorption of PS MPs and PS NPs by the magnetic mFMC composite was investigated over the pH range of 2–11 and the results are presented in Fig. 9(a). PS MP/NP removal efficiencies are significantly dependent on the pH of the solution. High removal efficiencies were obtained under acidic and neutral pH conditions, while a significant decrease in adsorption performance was observed under alkaline conditions. After 360 minutes of contact time, the removal efficiencies obtained for MP under pH conditions of 2, 5, and 7 were approximately 73.2%, 86.8%, and 93.0%, respectively. Similarly, for NP under the same pH conditions, the removal efficiencies were calculated as 78.0%, 81.5%, and 92.5%, respectively. The results show that the highest adsorption efficiency for both MP and NP occurred under neutral conditions (approximately pH 7). In contrast, increasing the pH to the alkaline region resulted in a significant decrease in adsorption efficiency. Specifically, even after 720 minutes at pH 11, the MP and NP removal efficiencies remained at only 11.5% and 8.0%, respectively. This is explained by the pH dependent change in the adsorbent surface chemistry. Under high pH conditions, the deprotonation of oxygen-containing functional groups such as –COOH and –OH on the mFMC composite surface causes the surface to acquire a more negative charge.^115,121^ As shown in Fig. 9(b), the zeta potential results also support this interpretation, showing that the surface charge of the mFMC composite decreased from +18.73 mV at pH 2 to −27.32 mV at pH 11. Similarly, it was determined that the surface charge of PS particles also becomes more negative, especially under alkaline conditions. Therefore, the increase in electrostatic repulsion between the adsorbent and PS MP/NP particles under high pH conditions has been effective in reducing adsorption efficiency. It is further suggested that the competition between OH^−^ ions, present at high concentrations in alkaline environments, and PS-MP/NP particles for the active binding sites on the adsorbent surface also contributes to this decrease.^122,123^

The relatively high removal efficiencies observed between pH 2 and 7 indicate that PS MP/NP adsorption was not controlled by electrostatic interactions alone. In addition to surface charge effects, hydrophobic interactions and π–π interactions may contribute to the attachment of PS particles to the carbon-rich regions of mFMC. Xing *et al.*^124^ reported that electrostatic interactions, n–π interactions and π–π interactions jointly contributed to the adsorption of PS nanoplastics onto ZnCl_2_-modified activated carbon. Similarly, FTIR band shifts reported for FeCl_3_-activated lignin-based carbon were attributed to π–π interactions between the aromatic surface of the adsorbent and the benzene rings of PS nanoplastics.^125^ These findings support the contribution of the π-conjugated CNT domains to PS attachment at the mFMC-water interface. However, the sharp decline in removal efficiency at pH 11 suggests that electrostatic repulsion became dominant under strongly alkaline conditions and weakened the contribution of these non-electrostatic interactions. A comparable decrease in PS nanoplastic adsorption with increasing pH has been directly attributed to stronger electrostatic repulsion between negatively charged PS particles and the adsorbent surface.^126^ Overall, the pH dependent adsorption behavior reflects the combined influence of surface charge, hydrophobic interactions and π–π interactions at the mFMC-water interface.

#### 3.2.4 DLVO analysis of pH-dependent interfacial interactions

**Fig. 10:**
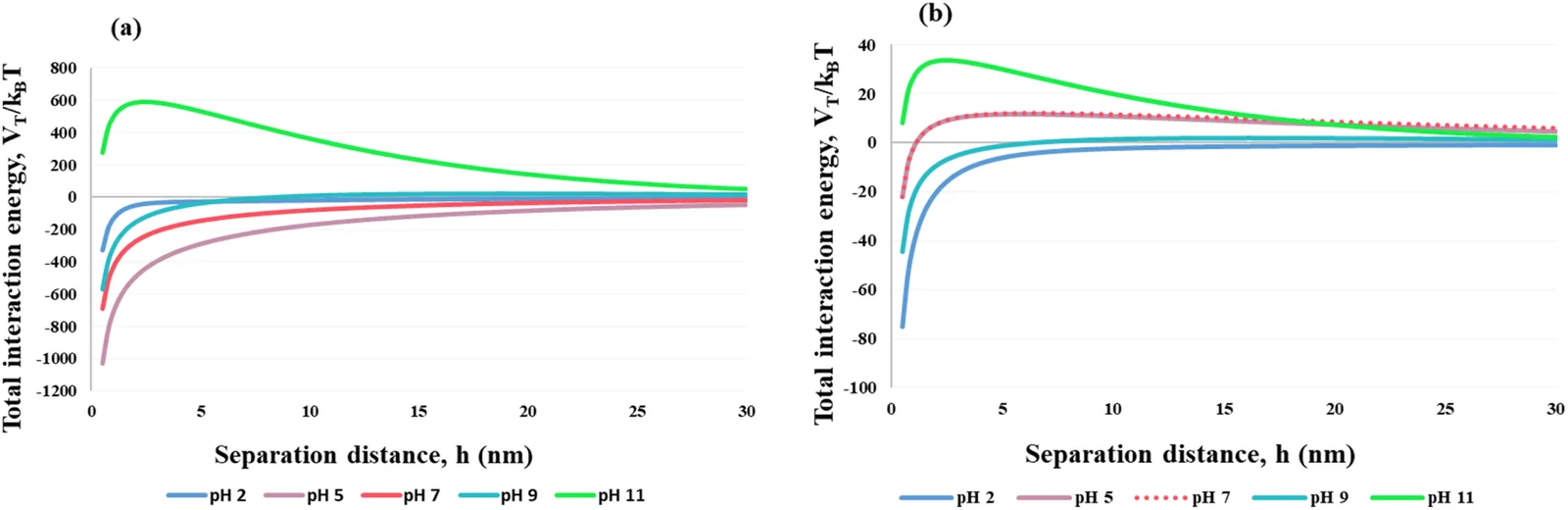
Total DLVO interaction-energy profiles between mFMC and (a) PS MPs and (b) PS NPs at pH 2, 5, 7, 9 and 11.

The pH dependent total interaction-energy profiles calculated for the interactions of mFMC with PS MPs and PS NPs are presented in Fig. 10 while the corresponding maximum energy barriers, barrier positions and experimental removal efficiencies are summarized in Table 1. Consistent with the approximations outlined in Section 2.5, the calculated barrier magnitudes reflect qualitative trends across pH conditions rather than precise absolute values. The calculations revealed marked differences between the two particle fractions, arising from their distinct zeta potentials, particle radii and electrostatic interactions with the mFMC surface.

**Table 1:** Classical DLVO parameters and calculated interaction-energy barriers for mFMC-PS MP and mFMC-PS NP pairs at different pH values

pH	Interaction pair	KCl-equivalent ionic strength (M)	Debye length (nm)	Maximum interaction-energy barrier (*V*_max_/*k*_B_*T*)	Separation distance at *V*_max_ (nm)	Removal efficiency (%)
2	mFMC-MP	0.02304	2.00	No positive barrier	—	73.2
2	mFMC-NP	0.02304	2.00	No positive barrier	—	78.0
5	mFMC-MP	0.000243	19.50	No positive barrier	—	86.8
5	mFMC-NP	0.000243	19.50	11.75	5.84	81.5
7	mFMC-MP	0.000171	23.27	No positive barrier	—	93.0
7	mFMC-NP	0.000171	23.27	11.98	6.38	92.5
9	mFMC-MP	0.000248	19.32	22.71	18.85	17.1
9	mFMC-NP	0.000248	19.32	1.86	16.08	15.6
11	mFMC-MP	0.001017	9.54	589.06	2.45	4.0
11	mFMC-NP	0.001017	9.54	33.67	2.47	2.0

At pH 2, both mFMC and PS MPs exhibited positive zeta potentials. Their like signed charges therefore generated a repulsive electrostatic double-layer contribution. However this repulsion was outweighed by the attractive van der Waals contribution and no positive total interaction energy barrier was predicted for the mFMC-MP pair. The relatively high ionic strength at this pH also compressed the electrical double layer, limiting the range of electrostatic repulsion. By contrast, mFMC and PS NPs exhibited oppositely signed zeta potentials, producing an attractive electrostatic contribution and likewise preventing the formation of a positive total interaction-energy barrier. As shown in Table 1 and Fig. 10, no positive barrier was obtained for either particle fraction at pH 2. This barrier free interaction was consistent with the relatively high removal efficiencies observed after 360 min, namely 73.2% for PS MPs and 78.0% for PS NPs. The influence of surface charge and electrolyte conditions on the interaction and aggregation behaviour of PS nanoplastics has similarly been demonstrated through zeta potential measurements and DLVO calculations.^127,128^

At pH 5, mFMC was negatively charged, whereas PS MPs retained a positive zeta potential. The resulting electrostatic attraction prevented the formation of a positive total interaction-energy barrier for the mFMC-MP pair, which was consistent with the high MP removal efficiency of 86.8%. In contrast, both mFMC and PS NPs were negatively charged at this pH, resulting in electrostatic repulsion and a calculated maximum interaction-energy barrier of 11.75 *k*_B_*T* at a separation distance of 5.84 nm. Despite this finite barrier, the NP removal efficiency remained high at 81.5%, indicating that the attachment process was not governed solely by the classical balance between electrostatic double layer and van der Waals interactions. Xing *et al.*^124^ demonstrated that PS nanoplastic adsorption onto ZnCl_2_ modified activated carbon involved the combined contributions of pore filling, electrostatic interactions and n–π and π–π interactions. Similarly, Shi *et al.*^129^ reported that the adsorption of differently functionalized PS nanoplastics onto magnetic biochars was controlled by the combined effects of hydrophobic interactions, electrostatic forces, hydrogen bonding and π–π interactions. These findings may support the contribution of additional short range interactions to NP attachment on the carbon rich and heterogeneous mFMC surface under conditions where the classical DLVO model predicts a moderate repulsive barrier.

A similar difference between the MP and NP interaction profiles was observed at pH 7. The slightly positive zeta potential of PS MPs and the negative zeta potential of mFMC produced an attractive electrostatic interaction and no positive total interaction-energy barrier was obtained for the mFMC-MP pair. Accordingly, the highest MP removal efficiency of 93.0% was observed at this pH. In contrast, both mFMC and PS NPs exhibited negative zeta potentials, giving rise to a calculated maximum interaction-energy barrier of 11.98 *k*_B_*T* at a separation distance of 6.38 nm. Nevertheless, PS NP removal reached 92.5% after 360 min. This apparent discrepancy suggests that the classical DLVO components did not fully represent the interactions occurring at the heterogeneous mFMC-water interface. Ren *et al.*^127^ showed that the environmental behaviour of PS nanoplastics may be affected not only by electrostatic double-layer and van der Waals forces but also by non-DLVO interactions, including hydrogen bonding, π–π interactions and bridging effects. Furthermore, Liu and Wang^13^ achieved high PS removal using CNT-modified aromatic adsorbents and attributed the enhanced attachment to the combined effects of electrostatic attraction, improved aqueous dispersion and π–π interactions between aromatic structures. These observations may be consistent with the contribution of CNT-rich domains and other heterogeneous adsorption sites to the high PS removal obtained at neutral pH.

At pH 9, mFMC, PS MPs and PS NPs all exhibited negative zeta potentials, creating electrostatically unfavourable conditions for adsorption. For the mFMC-MP pair, this repulsion generated a maximum interaction-energy barrier of 22.71 *k*_B_*T* at a separation distance of 18.85 nm, which was consistent with the pronounced decrease in MP removal efficiency to 17.1%. For PS NPs, a lower maximum barrier of 1.86 *k*_B_*T* was obtained at 16.08 nm. This difference was primarily related to particle size because the interaction energy in the HHF approximation scales with particle radius.^60,130^ However, the lower pairwise barrier for PS NPs should be considered together with the competitive conditions of the binary suspension. The comparison of the single and binary component systems showed that the smaller and more mobile PS NPs could reach accessible interfacial regions earlier and influence the subsequent availability of adsorption sites for PS MPs. Under the alkaline conditions at pH 9, where the negatively charged mFMC surface already provided fewer energetically favourable attachment regions, competition for the remaining accessible interface was likely intensified. Consequently, the simultaneous presence of PS MPs and PS NPs may have restricted the effective attachment opportunities of both particle fractions, resulting in similarly low removal efficiencies of 17.1% and 15.6%, respectively. Therefore, the behaviour observed at pH 9 can be interpreted as the combined outcome of DLVO-predicted electrostatic repulsion and size-dependent competitive adsorption within the mixed PS MP/NP system.

The strongest electrostatic inhibition was observed at pH 11, where mFMC, PS MPs and PS NPs all exhibited strongly negative zeta potentials. Under these conditions, the DLVO calculations predicted a very high maximum interaction-energy barrier of approximately 5.9 × 10^2^*k*_B_*T* for the mFMC-MP pair at a separation distance of 2.45 nm. A substantial positive barrier of 33.67 *k*_B_*T* was also obtained for the mFMC-NP pair at 2.47 nm. These results demonstrate that the simultaneous approach of both PS particle fractions to the negatively charged mFMC surface was strongly hindered by electrostatic repulsion. Importantly, the high barriers remained evident throughout the investigated Hamaker constant range, confirming that the interpretation was not dependent on a single selected value. The DLVO predictions were in close agreement with the experimental results as the removal efficiencies decreased to only 4.0% for PS MPs and 2.0% for PS NPs after 360 min. Similar decreases in PS nanoplastic adsorption under alkaline conditions have been attributed to the increasing electrostatic repulsion between negatively charged plastic particles and negatively charged carbonaceous adsorbent surfaces.^126,131^ Therefore, the pH 11 results provide strong evidence that electrostatic repulsion became the dominant interfacial factor and effectively suppressed the attachment of both PS MPs and PS NPs to mFMC.

#### 3.2.5 The effect of temperature

To evaluate the effect of temperature on the adsorption of PS-MP/NP mixtures by the magnetic mFMC composite, experiments were conducted at 25, 35, and 45 °C. The results demonstrated that temperature had no significant effect on adsorption efficiency within the investigated temperature range. After 360 minutes, the removal efficiencies for MP were determined as 93%, 90%, and 92% at 25, 35, and 45 °C, respectively, while for NP these values were calculated as 92.5%, 91.3%, and 90.8%, respectively. These findings show that similar removal efficiencies were obtained at all three temperatures and that the adsorption process was not significantly affected by temperature. Similarly, in the C@FeO nanopillars/2D-MOF system reported by Haris *et al.*,^131,132^ high removal efficiencies were maintained despite a slight decrease in MP removal with increasing temperature.

#### 3.2.6 Adsorption in real samples

**Fig. 11:**
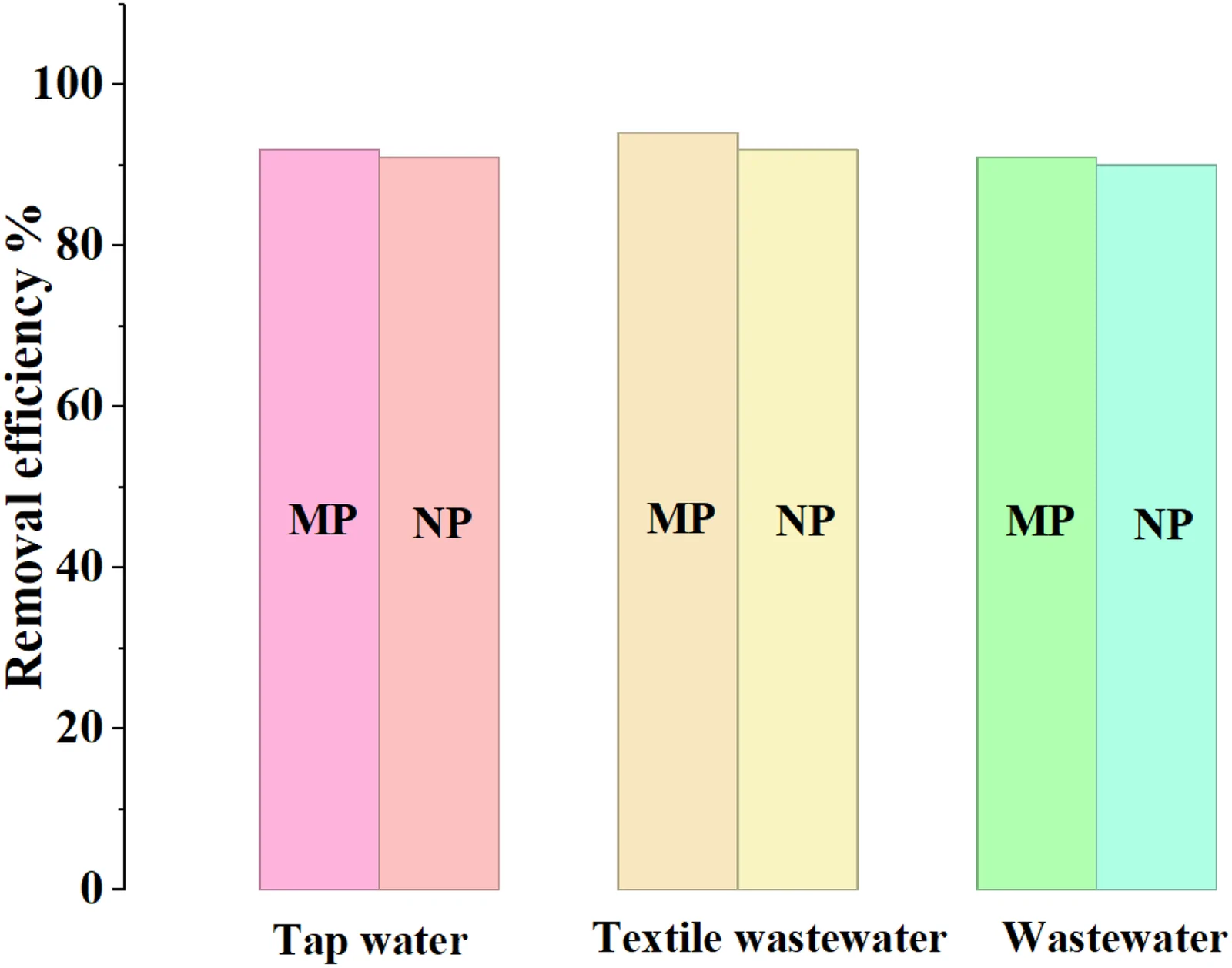
Simultaneous MP/NP removal efficiencies in tap water, textile wastewater and wastewater (total MP/NP concentration: 100 µg mL^−1^, mFMC dosage 2.5 mg initial pH values: 7.2, 6.9 and 7.3, respectively).

To evaluate the practical applicability of the developed magnetic mFMC composite, three different real water matrices-tap water, textile wastewater, and urban wastewater treatment plant effluent-were used, and the resulting adsorption efficiencies are presented in Fig. 11. Unlike pure water solutions prepared under laboratory conditions, the real water matrices retained their dissolved organic and inorganic constituents, including natural organic matter and various ions, following the standard 0.45 µm pre-filtration step applied prior to testing, thereby providing an assessment of adsorption performance under representative water quality conditions. Therefore, comparing the removal efficiency in real water matrices with optimized conditions is a critical indicator of the practical applicability of the adsorbent. The results obtained after 360 minutes of contact time reveal that the magnetic mFMC composite exhibits high adsorption efficiencies even in real water matrices. The three real water matrices exhibited initial pH values of 7.2 (tap water), 6.9 (textile wastewater) and 7.3 (wastewater treatment plant effluent), with corresponding electrical conductivities of 525, 1640 and 845 µS cm^−1^, respectively. Removal efficiencies for MP and NP in tap water were determined to be 92% and 91%, respectively; 94% and 92% in textile wastewater; and 91% and 90% in urban wastewater treatment plant effluent. These values are quite close to the removal efficiencies of 93% MP and 92.5% NP obtained in the pure system under optimized conditions, and high removal efficiencies of 90% and above were achieved in all matrices. These results show that the composite maintains strong and consistent adsorption performance even in complex water conditions, regardless of the type of water matrix.

#### 3.2.7 Adsorption mechanism

When comparing the pre- and post-adsorption FTIR spectra of the mFMC composite (Fig. S2), the characteristic bands of the composite were generally preserved. However, shifts and decreases in peak intensities occurred in some vibrational bands. The difference observed particularly in the 1450–1500 cm^−1^ region is consistent with the approximately 1492 cm^−1^ aromatic ring vibrational band of PS reported in the literature.^133,134^ This supports the idea that PS interacts with the mFMC surface and is adsorbed onto the composite surface. These changes indicate that interaction occurs between MP/NP and mFMC surface functional groups, while the structural integrity of the composite is preserved.

**Fig. 12:**
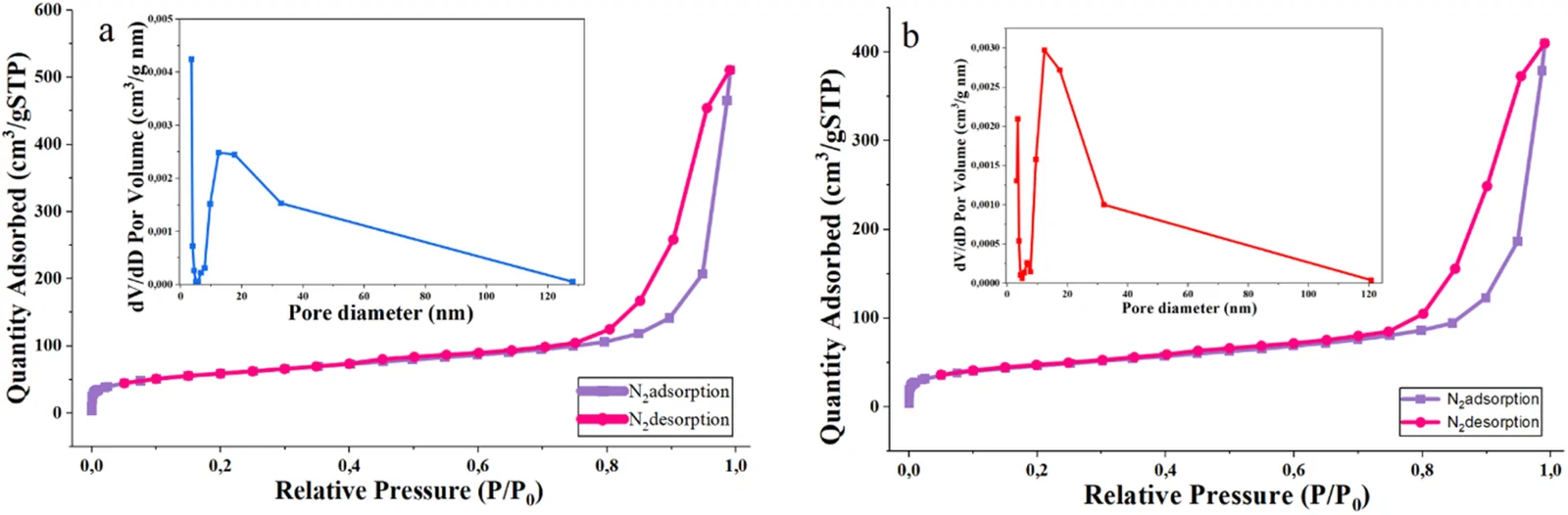
N_2_ adsorption–desorption isotherms obtained before (a) and after (b) adsorption.

When the N_2_ adsorption–desorption isotherms obtained before (a) and after (b) adsorption in Fig. 12 are examined, it is seen that both samples exhibit Type IV isotherm behavior with a distinct hysteresis loop. This indicates that the material has a mesoporous structure. While a rapid increase in adsorption was observed in the low relative pressure region in the pre-adsorption sample, a significant increase in the amount of adsorbed gas occurred at high relative pressure values due to capillary condensation. When the isotherm obtained after adsorption is examined, it is seen that the isotherm shape is generally preserved, but there is a slight decrease in the amount of adsorbed gas in the high relative pressure region. This indicates that a portion of the accessible pore volume after the adsorption process is filled by the adsorbed MP/NPs. The decrease in BET surface area from 205 m^2^ g^−1^ to 161 m^2^ g^−1^ and BJH cumulative pore volume from 0.86 cm^3^ g^−1^ to 0.72 cm^3^ g^−1^ also supports this finding. The BET surface area obtained in this study was found to be higher compared to the values reported for some similar magnetic MOF composites in the literature, such as Fe_3_O_4_@CMC-MIL-101-NH_2_ (199.5 m^2^ g^−1^)^135^ and Fe_3_O_4_@MIL-100(Fe) (104.13 m^2^ g^−1^).^136^ This result demonstrates that the mFMC composite possesses an enhanced surface area and pore structure capable of supporting adsorption. Furthermore, the increase in the average pore diameter calculated using the BJH method from 4.5 nm to 13.3 nm can be explained by the shift in the measurable pore distribution towards larger pores, particularly as a result of the partial blockage of smaller pores by adsorbate molecules. The fact that the isotherm type does not change before and after adsorption indicates that the pore structure is largely preserved during the adsorption process and that the material is structurally stable. These results show that the adsorption process does not completely disrupt the pore structure of the adsorbent, but causes a partial reduction in the accessible surface area and pore volume.

**Fig. 13:**
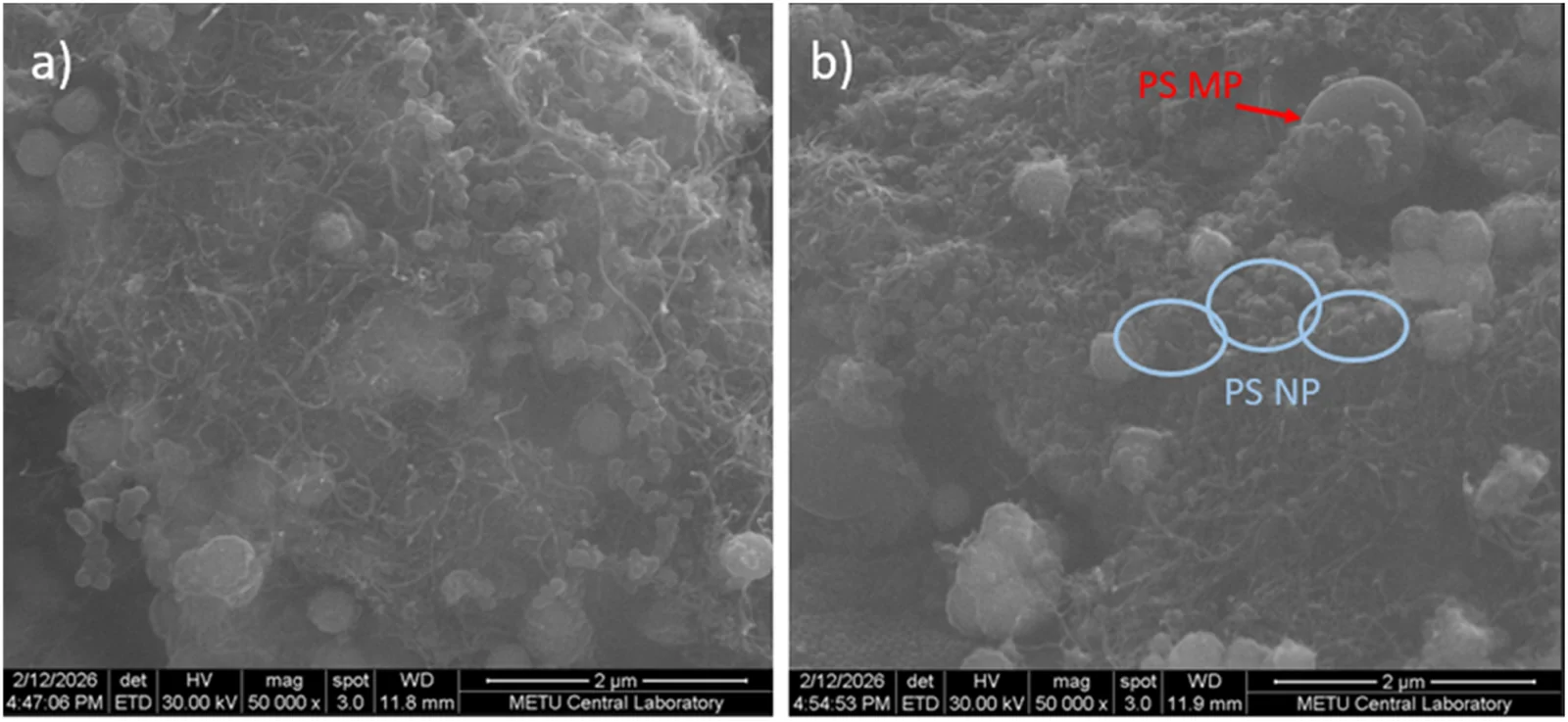
SEM images of mFMC before and after adsorption: (a) before adsorption and (b) after adsorption.

Comparing the pre- and post-adsorption SEM images presented in Fig. 13 shows that significant changes occurred in the structure of mFMC after adsorption. The observation of micrometer-sized spherical particles indicates that microplastics accumulated on the adsorbent surface. Furthermore, it is considered that small and clustered structures are related to nanoplastic adsorption. These findings demonstrate that mFMC exhibits effective adsorption performance in the removal of MP/NPs.

**Fig. 14:**
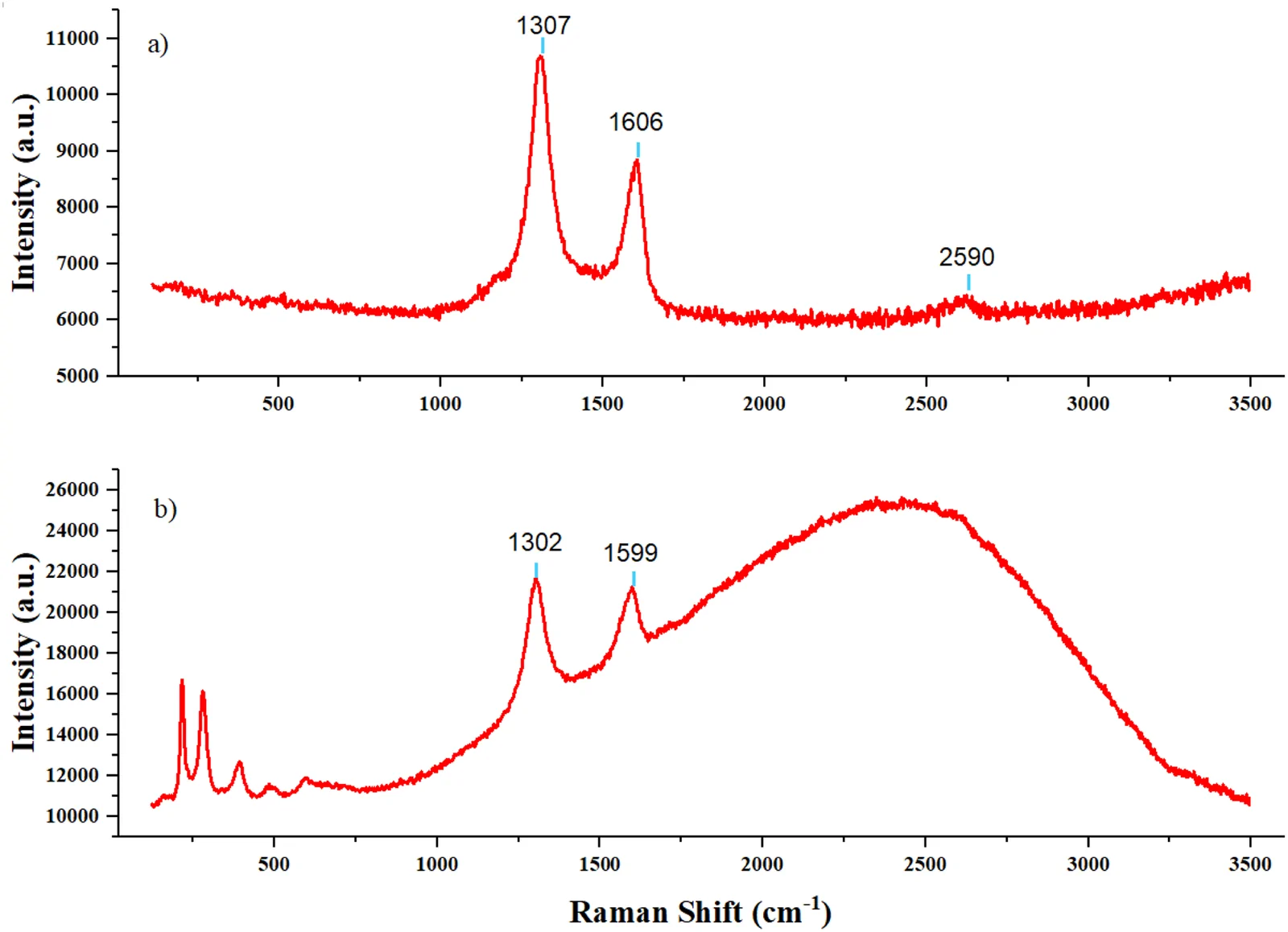
(a) Raman spectrum before adsorption, (b) Raman spectrum after adsorption.

In the pre-adsorption Raman spectrum of the mFMC composite presented in Fig. 14(a), two characteristic bands were observed: 1307 cm^−1^ (D band) and 1606 cm^−1^ (G band). The D band is associated with defects and irregularities in the carbon structure, while the G band represents the graphitic vibrational mode of sp^2^ hybridized carbon atoms.^137–139^ Furthermore, the band observed around 2590 cm^−1^ corresponds to the 2D band, a second-order Raman band commonly found in carbon nanotubes.^140,141^ These bands confirm the presence of carbon nanotubes within the composite structure. The *I*_D_/*I*_G_ ratio calculated before adsorption is 1.21, indicating the presence of suitable active sites for adsorption on the surface.^142^ Following the adsorption of PS MP/NP a slight shift of the D and G bands to values of 1302 cm^−1^ and 1599 cm^−1^, respectively, was observed (Fig. 14(b)). Furthermore, the *I*_D_/*I*_G_ ratio decreased to 1.02, indicating that the adsorbed MP/NP particles partially covered the active sites on the CNT surface. π–π interactions that can occur between the aromatic rings in the polystyrene structure and the graphitic surface of the CNT may play a significant role in this adsorption mechanism and explain the observed band shifts and ratio changes.^143^ The broad increase in intensity observed in the 2000–3000 cm^−1^ range in the post-adsorption spectrum is associated with the fluorescence background originating from fluorescent polystyrene particles. Such broad background signals have been commonly reported in Raman assays of fluorescent polymers and can partially mask Raman bands.^144^ Therefore, the observed spectral changes support the successful adsorption of MP/NP onto the composite surface.

#### 3.2.8 Evaluation of mFMC for PS MP/NP adsorption

**Fig. 15:**
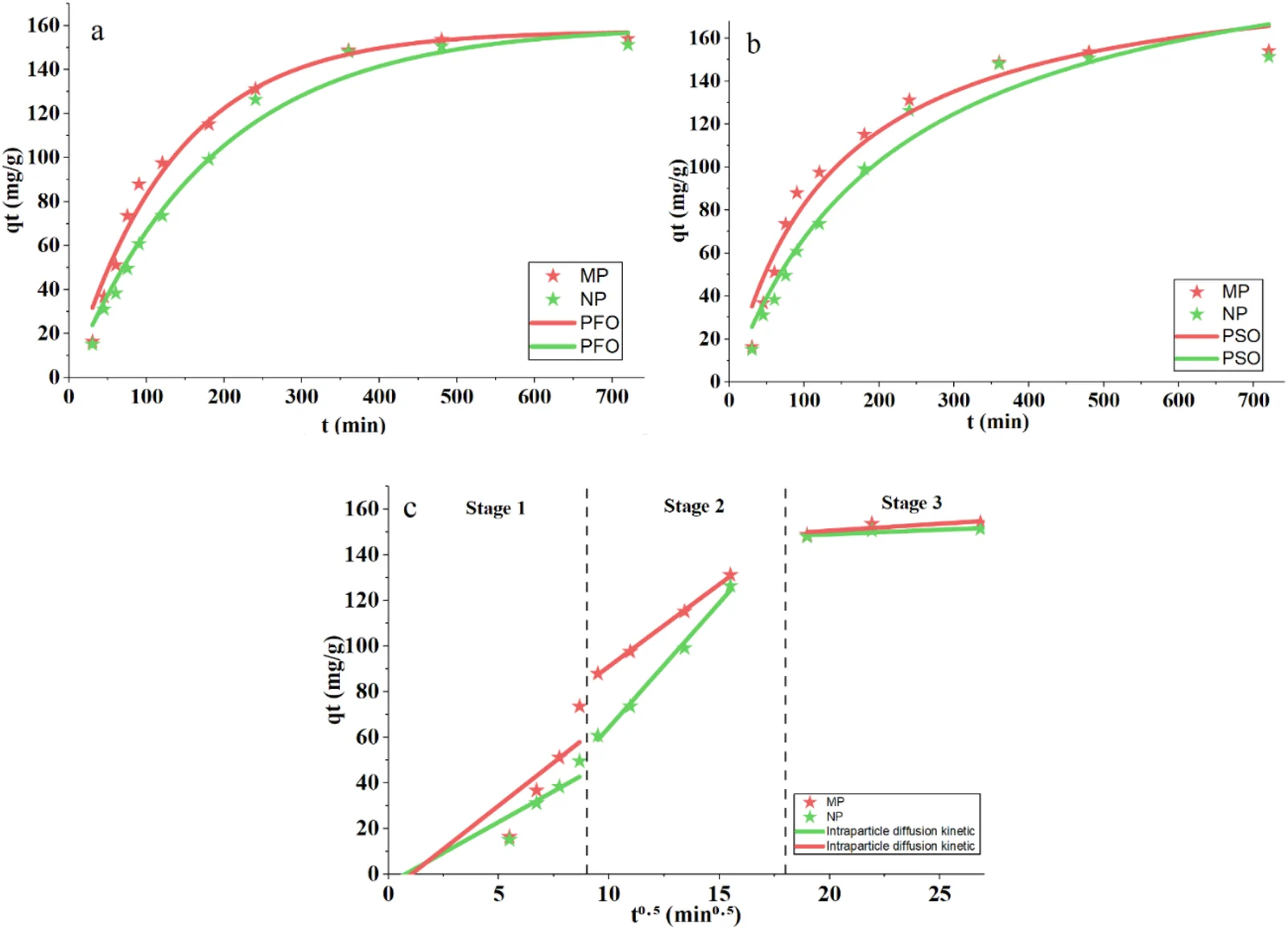
(a) PFO, (b) PSO and (c) Weber–Morris intraparticle diffusion kinetic model graphs for mFMC (temperature: 25 °C, total MP/NP concentration: 100 µg mL^−1^, mFMC dosage 2.5 mg pH 7).

To investigate the adsorption kinetics of PS MP and PS NP on mFMC composite in a PS MP/NP mixture, experimental data were analyzed using pseudo-first-order (PFO), pseudo-second-order (PSO), and Weber–Morris intraparticle diffusion models, and the results are presented in Fig. 15(a–c). In the initial stages of the adsorption process, the adsorption capacity for both MP and NP increased rapidly, then approached an equilibrium plateau. When the kinetic model fit results are examined, it is seen that the correlation coefficients of the PFO model (Fig. 15(a)) (MP: *R*^2^ = 0.978, NP: *R*^2^ = 0.985) are higher than those of the PSO model (MP: *R*^2^ = 0.960, NP: *R*^2^ = 0.969). Furthermore, the theoretical equilibrium capacities calculated from the PFO model (MP: 157.55 mg g; NP: 160.0 mg g^−1^) closely match the experimental *Q*_e_ values (MP: 154.08 mg g^−1^, NP: 151.36 mg g^−1^). In contrast, the theoretical *Q*_e_ values obtained from the PSO model (Fig. 15(b)) (MP: 197.37 mg g^−1^, NP: 218.58 mg g^−1^) deviate significantly from the experimental data. These results demonstrate that PS MP/NP adsorption on mFMC is better characterized by PFO kinetics, consistent with a rate-limiting step governed primarily by physical interactions.^145,146^ This interpretation is further supported by the zeta potential, DLVO and Raman analyses presented above, rather than resting on kinetic model fitting alone. Comparing the rate constants obtained from advanced fit calculations using the PFO model, it is observed that the calculated *K*_1_ value for MP (7.52 × 10^−3^ min^−1^) is significantly higher than the *K*_1_ value obtained for NP (5.40 × 10^−3^ min^−1^). This indicates that micro-sized particles make faster contact with the adsorbent outer surface and that the initial adsorption rate is higher for MP.^145^ To investigate the adsorption mechanism in more detail, an intraparticle diffusion model was applied. As shown in Fig. 15(c), the *q*_*t*_ – *t*_0.5_ plots exhibit three distinct linear regions (Stage 1, Stage 2, Stage 3) for both PS MP and PS NP. The fact that the graphs neither pass through the origin nor exhibit zero intercept indicates that intraparticle diffusion is not the only rate-determining step.^147^ Stage 1 represents the rapid surface adsorption occurring on the active binding sites on the outer surface of the mFMC. Stage 2 corresponds to the pore diffusion stage, where diffusion occurs into the adsorbent pores and extremely high *R*^2^ values were obtained for both systems (MP: 0.999, NP: 0.994). Stage 3 represents the equilibrium plateau where the active binding sites approach saturation. When the diffusion rate constant (*k*S_2_) values for Stage 2 are examined, it is seen that the value obtained for NP (10.904 mg g^−1^ min_0.5_) is significantly higher than the value calculated for MP (7.202 mg g^−1^ min_0.5_). This result indicates that NPs can diffuse into the internal pore structure of mFMC more quickly due to their smaller dimensions.^148^

Isotherm data for MP and NP adsorption were analyzed using both Langmuir and Freundlich models. Isotherm plots for MP/NP are presented in Fig. S3(a and b). The Langmuir model showed a very high agreement for MP with *R*^2^ = 0.963 and for NP with *R*^2^ = 0.969; however, the Freundlich model yielded much lower *R*^2^ values for both pollutants (MP: 0.538, NP: 0.600). These results indicate that the adsorption process is in good agreement with the Langmuir isotherm model and suggesting that adsorption occurs *via* a monolayer mechanism on a homogeneous surface.^135^

#### 3.2.9 Reusability

**Fig. 16:**
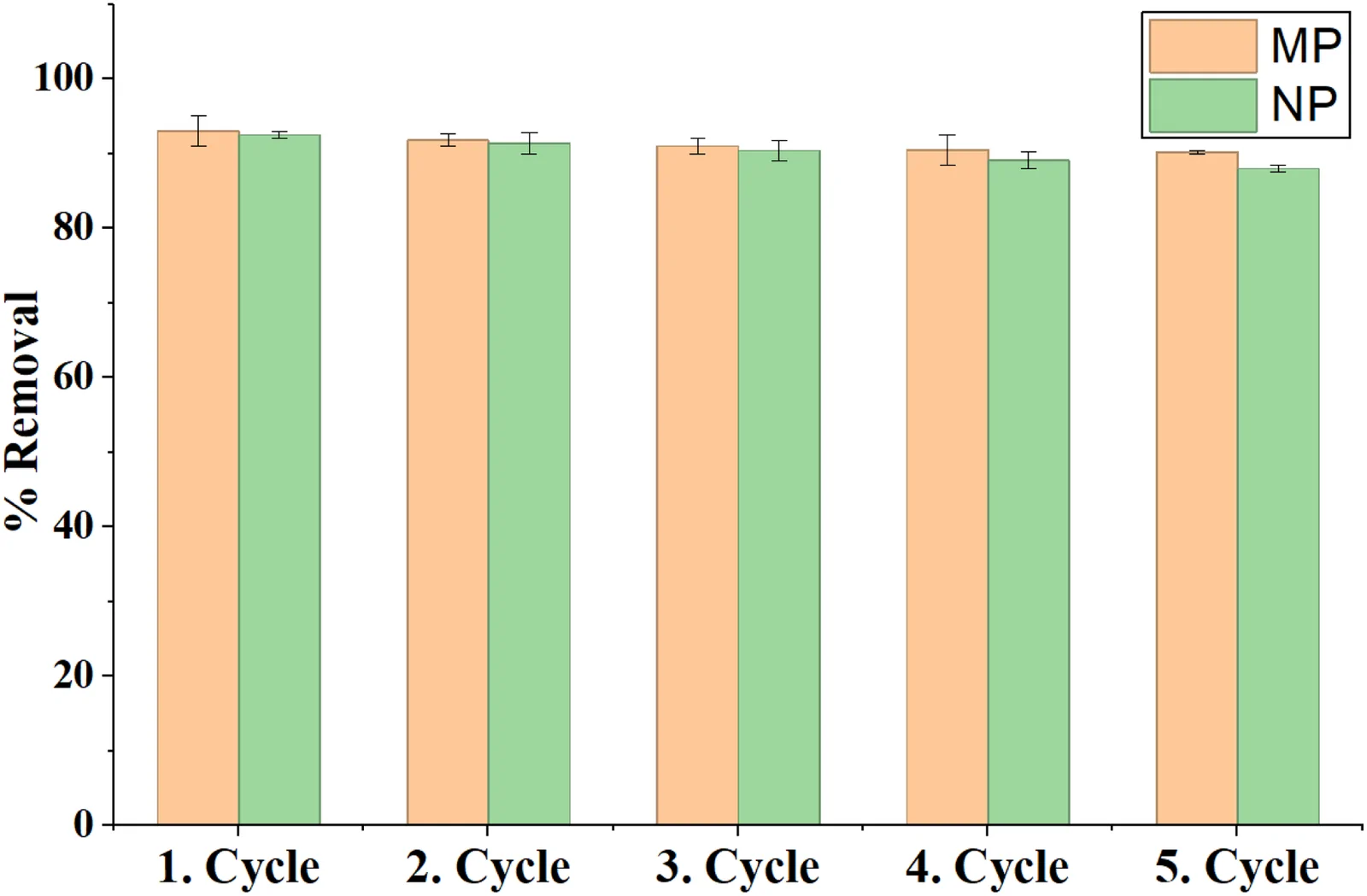
Reusability of mFMC for five cycles in MP/NP removal (temperature: 25 °C, total MP/NP concentration: 100 µg mL^−1^, mFMC dosage 2.5 mg pH 7, contact time: 360 min per cycle).

Reusability is considered a critical performance criterion for the economic and environmental sustainability of adsorbents in practical applications. Accordingly, the reusability of magnetic mFMC composite was investigated over five consecutive adsorption–desorption cycles, and the results are presented in Fig. 16.

MP removal efficiency decreased from 93.0% in the first cycle to 90.2% in the fifth cycle, while NP removal efficiency decreased from 92.5% to 88.0%. This gradual and limited decrease observed over five cycles can be attributed to the partial blockage of active binding sites during repeated adsorption–desorption processes or limited changes in the structural integrity of the mFMC composite. Maintaining high removal efficiencies even after five cycles indicates good structural stability and regeneration capacity of the composite over the tested cycle range. In comparison with similar studies, Zhou *et al.*^56^ reported that the UiO-66/MF composite showed an efficiency of over 81% after 25 cycles, while Feng *et al.*^135^ reported that magnetic MOF composites maintained an efficiency of >89.0% after five cycles.

## 4 Conclusion

The coexistence of MP/NP in aquatic systems creates complex adsorption behavior because particles of different sizes may compete for the same interfacial regions. Understanding these interactions is therefore essential for designing effective materials for their simultaneous removal. This study demonstrates that the adsorption of PS MP/NP on mFMC is controlled not only by removal capacity but also by particle-size-dependent access to the adsorbent–water interface. The comparison of single and binary component systems showed that the presence of PS NPs altered the early stage adsorption behavior of PS MPs, highlighting the importance of evaluating mixed particle systems rather than predicting their behavior from single component experiments. The combined zeta potential and DLVO results established surface charge and pH dependent interaction energy barriers as key factors governing particle attachment, particularly under alkaline conditions. Surface characterization further indicated that PS accumulation occurred mainly over accessible external and CNT rich interfacial regions, with electrostatic and non-electrostatic interactions contributing to retention. The preservation of high removal in different water matrices and after repeated use also supports the stability and recoverability of the magnetic hybrid. Overall, mFMC provides a useful heterogeneous interface for clarifying how particle size, surface charge and competitive access influence the simultaneous capture of MP/NP. Future studies should examine environmentally aged particles, additional polymer types and the effects of natural organic matter to further establish the behavior of the system under environmentally relevant conditions.

## Author contributions

Meric Yilmaz Salman: conceptualization, methodology, investigation, formal analysis, writing – original draft. Türker Salman: conceptualization, methodology, investigation, writing. Mustafa Ersin Pekdemir: conceptualization, methodology, investigation, writing. All authors read and approved the final manuscript.

## Conflicts of interest

There are no conflicts to declare.

## Supplementary Material

RA-OLF-D6RA07127A-s001

## Data Availability

The original contributions presented in this study are included in the article and its supplementary information (SI). Further inquiries can be directed to the corresponding author. Supplementary information is available. See DOI: https://doi.org/10.1039/d6ra07127a.
