# Supplementary material for: Size dependent competitive adsorption of polystyrene micro and nanoplastics on a magnetic Fe3O4@MIL-100(Fe)/CNT hybrid: interfacial mechanisms and water treatment performance
Source: RSC Adv. 2026 Sep 14;16(44):54839–61. doi: 10.1039/d6ra07127a (PMC13573160; doi:10.1039/d6ra07127a)
Supplement: Supplementary file 1 [file RA-016-D6RA07127A-s001.pdf]

Supplementary Information (SI) for RSC Advances.
This journal is © The Royal Society of Chemistry 2026

Size dependent competitive adsorption of polystyrene micro and nanoplastics on a magnetic Fe_3O_4@MIL-100(Fe)/CNT hybrid: interfacial mechanisms and water treatment performance

Meric Yilmaz Salman[a], Türker Salman[b], Mustafa Ersin Pekdemir[c]*

[a]Keban Vocational School, Keban, Firat University, Elazığ, Türkiye

[b]Department of pharmacy services, Kovancılar Vocational School, Firat University, Elazığ, Türkiye

[c]Polymer and Biomaterials Research Laboratory, Department of Chemistry, Faculty of Science, Firat University, Elazığ, Türkiye

*Corresponding authors:

E-mail address: meric.yilmaz@firat.edu.tr (M. Yilmaz Salman)

Contents of the Supporting Information

1. Calculation of removal efficiency and adsorption capacity for MP/NP

2. DLVO interaction-energy calculations

2.1. Experimental inputs and representation of the ionic environment

Table S1. Experimental inputs and derived electrostatic parameters used in the DLVO calculations

2.2. Hamaker-constant sensitivity analysis

Table S2. Sensitivity of the maximum DLVO interaction-energy barriers to the effective Hamaker constant

3. Pseudo-first-order, pseudo-second-order and intraparticle diffusion models

4. Langmuir and Freundlich isotherm models

Fig. S1. The standard curve for aqueous solutions of MP (a) and NP (b)

Fig. S2. Pre- and post-adsorption FTIR spectra of the mFMC composite

Fig. S3. Langmuir and Freundlich isotherm plots for MP (a) and NP (b) adsorption

1. Removal efficiency =R= $(C_0\text{-Ce})/C_0$ x100 (S1)

Adsorption capacity= Qe= $[(C_0\text{-Ce})\text{x}V]/m$ (S2)

Where, Qe represents the equilibrium adsorption capacity per unit mass of mFMC composite (mg/g), C_0 and Ce represent the initial and equilibrium concentrations of PS MP/NP in the solution (µg/mL), respectively, V represents the total reaction volume (mL), and m represents the mass of mFMC composite used (mg).

2. DLVO interaction-energy calculations

The DLVO calculations were used to compare the relative favourability of PS MP and PS NP approach to the mFMC surface under different pH conditions.

The total interaction energy, V_T, was calculated as the sum of the electrostatic double layer interaction energy, V_{EDL} and the van der Waals interaction energy, V_{vdW}.

$V_T(h)= V_{EDL}(h)+ V_{vdW}(h)$ (S3)

The electrostatic double layer interaction energy was calculated as:

$V_{EDL}(h)= \pi\varepsilon_0\varepsilon_r R[(\psi_1^2+\psi_2^2) \ln(1-\exp(-2\kappa h))+2\psi_1\psi_2 \ln((1+\exp(-\kappa h))/(1-\exp(-\kappa h)))$ (S4)

where ε_0 is the vacuum permittivity, ε_r is the relative permittivity of water, R is the radius of the PS particle, ψ_1 and ψ_2 are the interaction potentials assigned to mFMC and the PS particle, respectively, κ is the inverse Debye length and his the surface to surface separation distance.

The non-retarded van der Waals interaction between a spherical PS particle and the locally planar mFMC surface was calculated as:

$V_{vdW}(h)= -A_{132}R/6h$ (S5)

where A_{132} is the effective Hamaker constant for the PS-water-mFMC system.

For a monovalent electrolyte, the inverse Debye length was calculated from the operational KCl-equivalent ionic strength:

$\kappa=((2e^2N_A(1000)I_{eq})/(\varepsilon_0\varepsilon_r k_B T))^{1/2}$ (S6)

where e is the elementary charge, N_A is Avogadro's constant, I_{eq} is the operational KCl-equivalent ionic strength expressed in mol L^{-1}, εr is the relative permittivity of water (78.5 at

25 ^{0}C), k_B is the Boltzmann constant and T is the absolute temperature. The factor of 1000 converts mol L^{-1} to mol m^{-3}.

2.1. Experimental inputs and representation of the ionic environment

The ionic environment was represented using conductivities measured directly in the equal-volume PS MP/NP binary suspensions at each pH. This ensured that the calculations reflected the same mixed-particle conditions used in the pH-dependent adsorption experiments.

The measured conductivities were converted to operational KCl-equivalent ionic strengths by linear scaling against the conductivity of a 0.01 M KCl reference solution at 25 ^{0}C:

I_{eq}= 0.01(σ/1.40823) (S7)

where σ is the measured suspension conductivity in mS cm^{-1} and 1.40823 mS cm^{-1} is the reference conductivity of the KCl solution.

Table S1. Experimental inputs and derived electrostatic parameters used in the DLVO calculations

pH	mFMC zeta potential (mV)	PS MP zeta potential (mV)	PS NP zeta potential (mV)	Binary-suspension conductivity (mS cm^{-1})	I_{eq} (M)	Debye length, κ^{-1} (nm)
2	+18.73	+8.73	−11.07	3.24400	0.023036	2.00
5	−16.81	+8.70	−15.43	0.03428	0.000243	19.50
7	−15.79	+2.00	−16.00	0.02406	0.000171	23.27
9	−5.77	−24.00	−17.75	0.03492	0.000248	19.32
11	−27.32	−45.00	−25.31	0.14320	0.001017	9.54

2.2. Hamaker-constant sensitivity analysis

Because the mFMC composite contains chemically different Fe_3O_4, MIL-100(Fe) and CNT-rich regions, the exact effective Hamaker constant of the PS-water-mFMC system cannot be represented by a single independently measured value. The principal calculations therefore used A_{132}=1x10^{-20} J, while the sensitivity of the calculated barriers was evaluated over the broader range of 0.5-3.0x10^{-20} J.

The principal pH-dependent trends remained unchanged over this range. In particular, the substantial interaction-energy barriers obtained for both PS particle fractions at pH 11 persisted throughout the sensitivity analysis. The moderate PS NP barriers at pH 5 and 7 also remained

positive, whereas the comparatively small PS NP barrier at pH 9 was more sensitive to the selected Hamaker constant.

Table S2. Sensitivity of the maximum DLVO interaction-energy barriers to the effective Hamaker constant

pH	Interaction pair	Barrier range over (k_BT)
5	mFMC-PS NP	6.85-13.81
7	mFMC-PS NP	7.49-13.86
9	mFMC-PS MP	4.43-28.35
9	mFMC-PS NP	0-2.55
11	mFMC-PS MP	460.52-635.12
11	mFMC-PS NP	22.02-38.52

3. The pseudo-first-order model

$$q_t = q_e\,(1-e^{-k_1 t}) \qquad (S8)$$

The pseudo second order model

$$q_t = \frac{q_e^2 k_2 t}{1 + q_e k_2 t} \qquad (S9)$$

Intraparticle diffusion models

$$q_t = k_p t^{0.5} + c \qquad (S10)$$

Where k_1 (min^{-1}) denotes the pseudo first order adsorption rate constant, k_2 ($g\cdot mg^{-1}\cdot min^{-1}$) represents the pseudo second order adsorption rate constant and kp ($mg\cdot g^{-1}\cdot min^{-1/2}$) stands for the intra particle diffusion adsorption rate constant. qe ($mg\cdot g^{-1}$) refers to the equilibrium adsorption capacity of the adsorbent, qt ($mg\cdot g^{-1}$) indicates the amount of MP/NP adsorbed onto the adsorbent at a specific contact time t (min). C is associated with the thickness of the boundary layer in the adsorption system.

4. Langmuir isotherm model:

$$q_e = \frac{q_m K_L C_e}{1 + K_L C_e} \qquad (S11)$$

Freundlich isotherm model:

$$q_e = K_F C_e^n \qquad \text{(S12)}$$

Where qe ($mg.g^{-1}$) and q_m ($mg\ g^{-1}$) are the equilibrium and maximum adsorption capacity of MP/NP, Ce ($mg\ L^{-1}$) is the equilibrium adsorption concentration; K_L (L/mg) is the Langmuir isothermal constant. n and KF [($mg\ g^{-1}$)·($L\ mg^{-1}$)1/n] are the Freundlich heterogeneity factor and empirical constant, respectively.

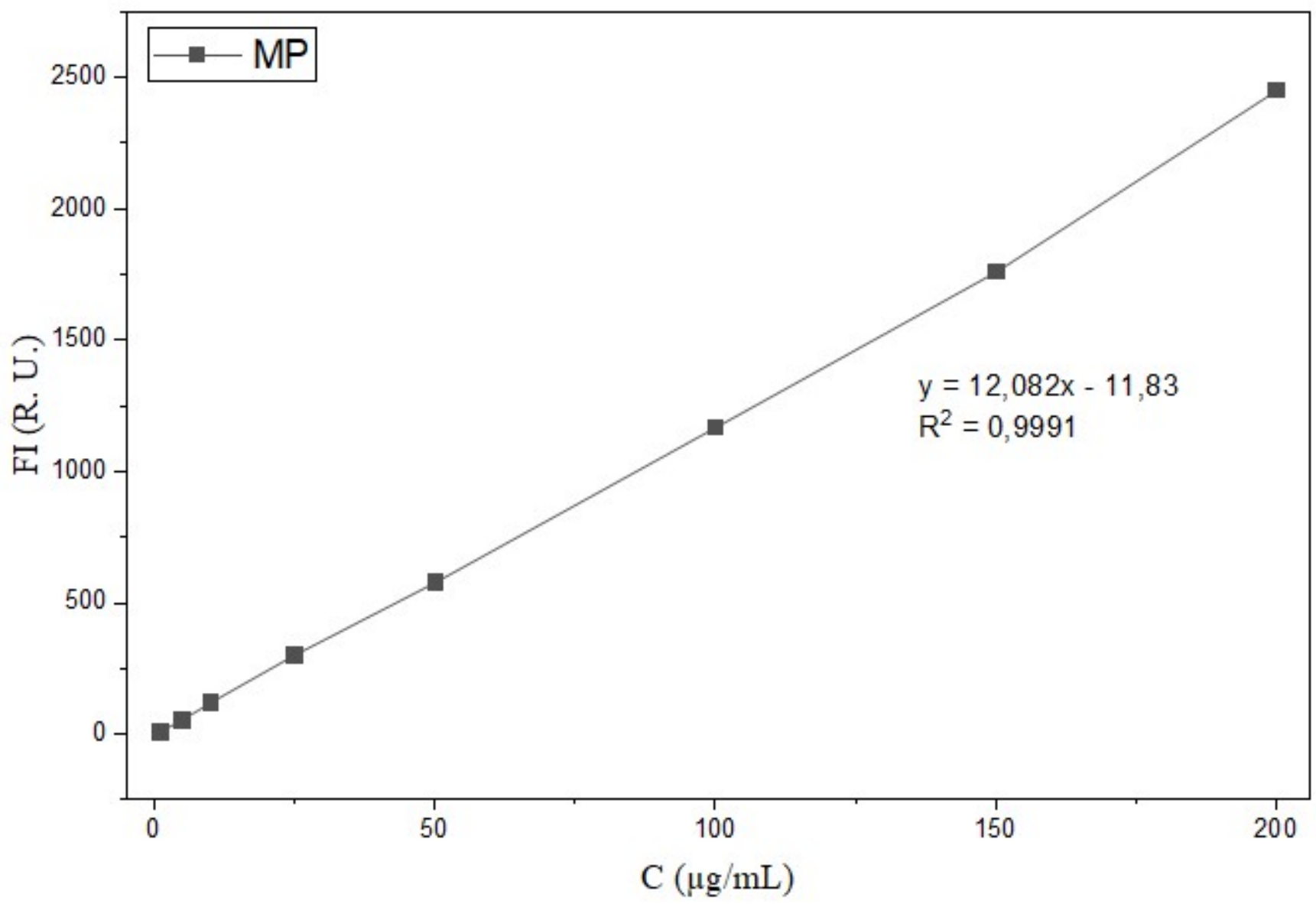

Fig. S1 (a). The standard curve for aqueous solutions of MP

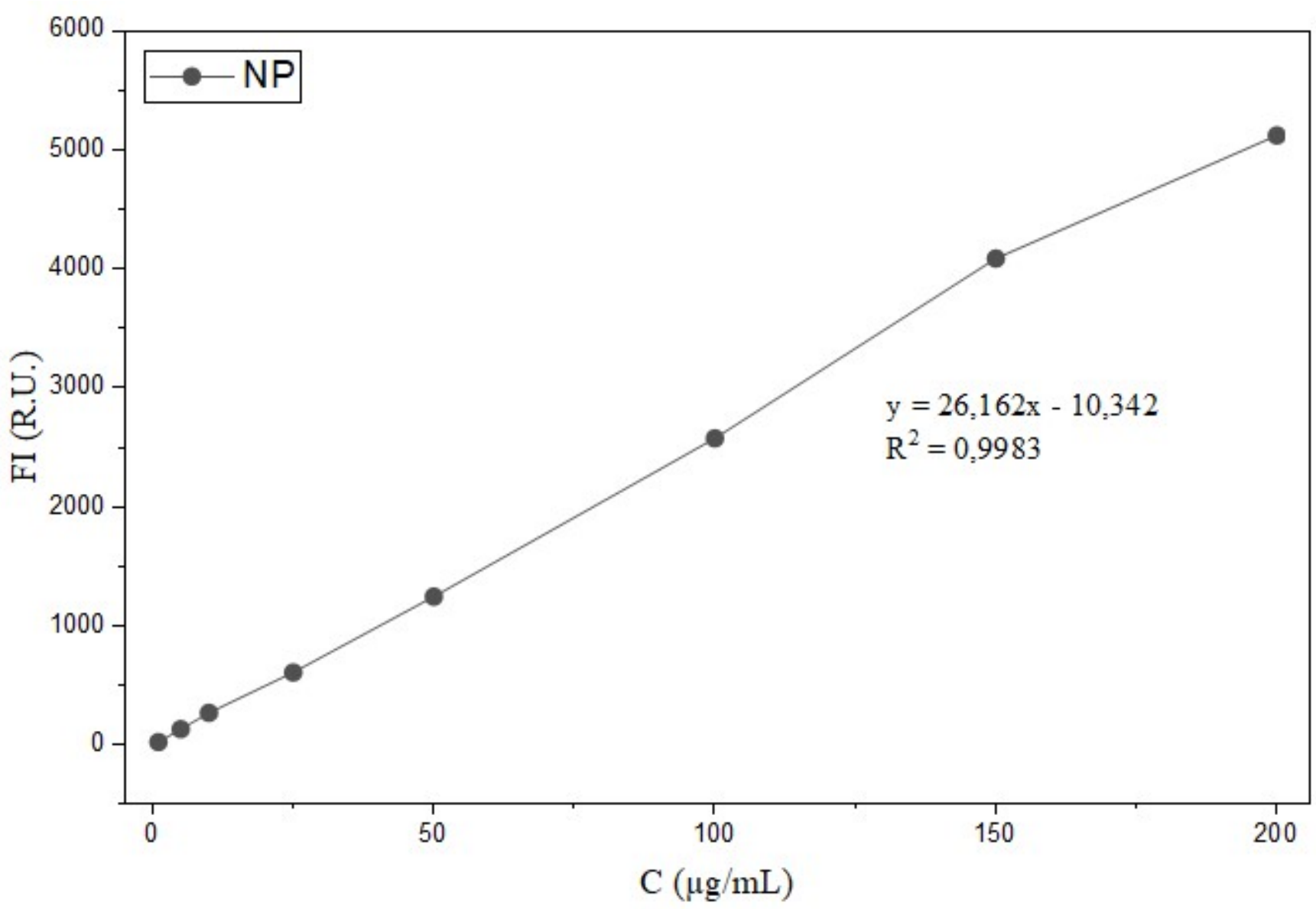

Fig. S1 (b). The standard curve for aqueous solutions of NP

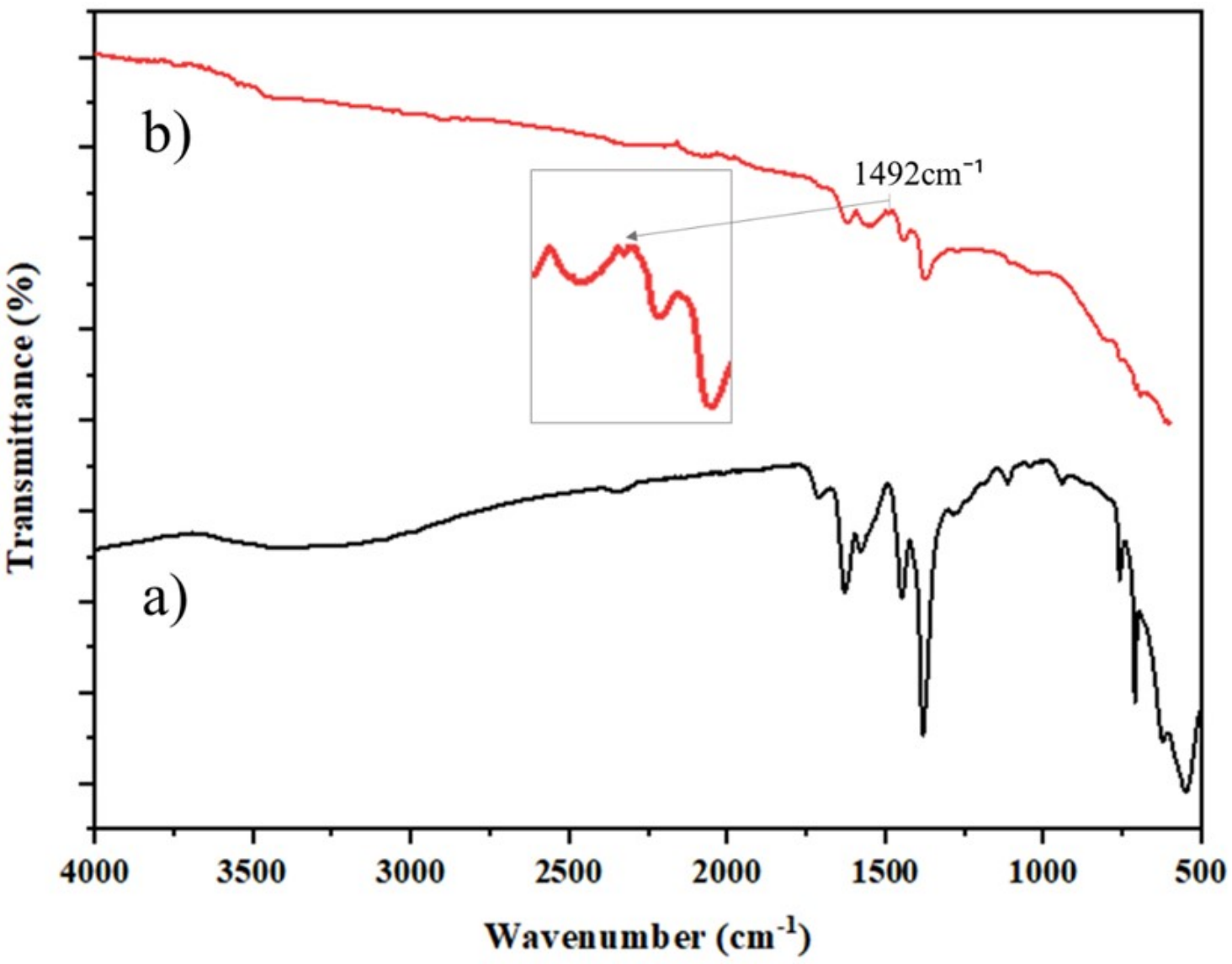

Fig. S2. Pre- and post-adsorption FTIR spectra of the mFMC composite

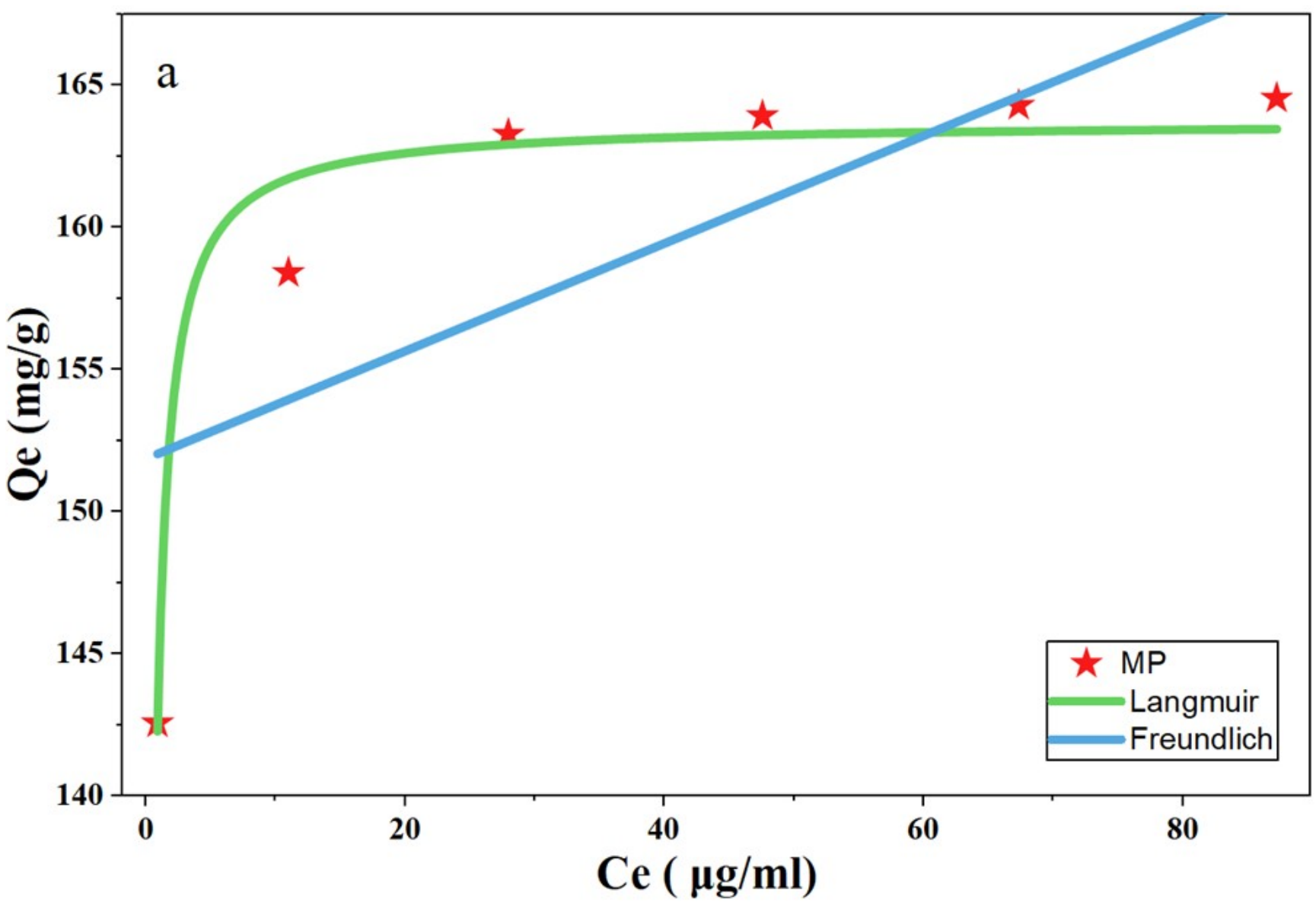

Fig. S3 (a). Langmuir and Freundlich isotherm plots for MP

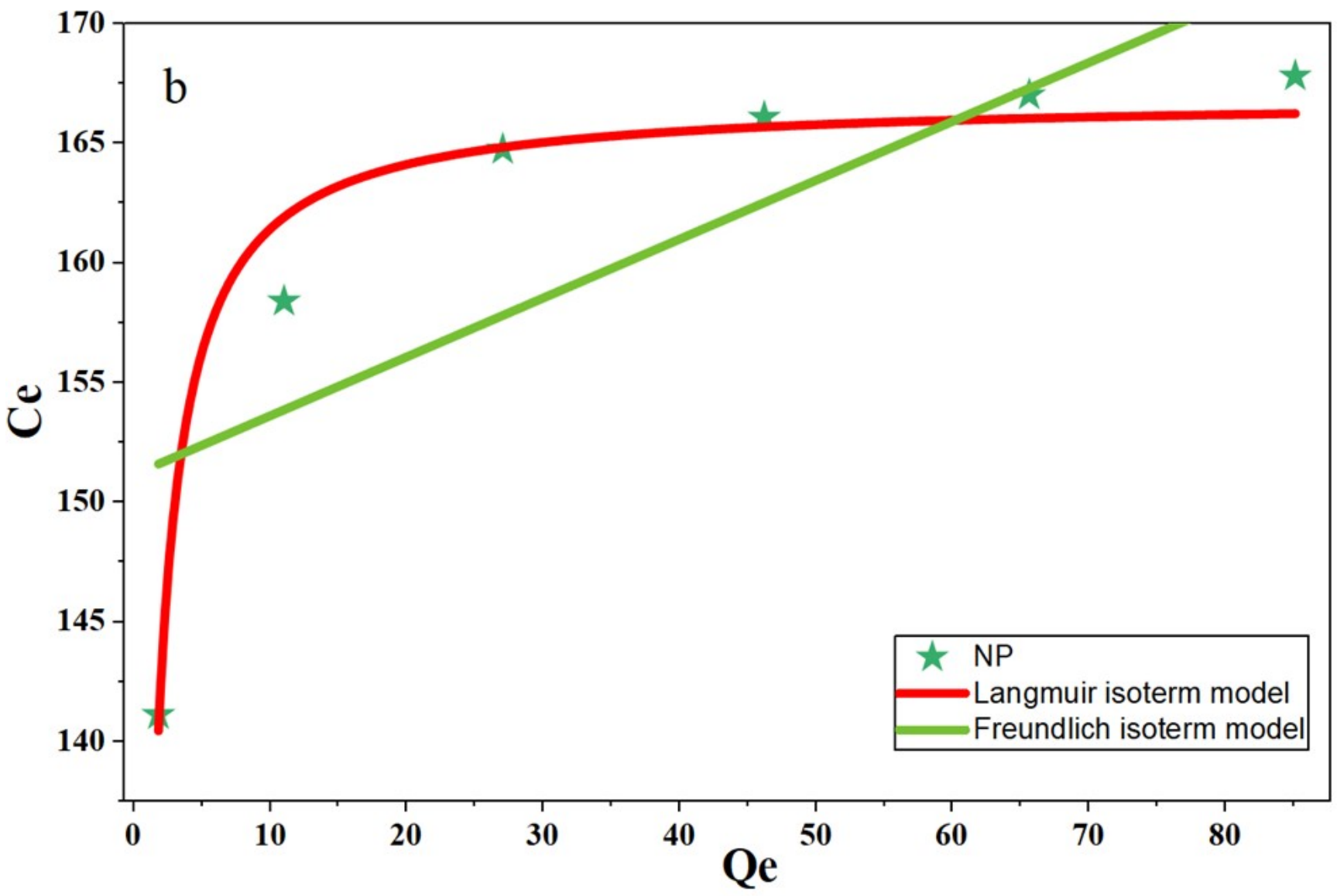

Fig. S3 (b). Langmuir and Freundlich isotherm plots for NP
